# Test equating sleep scales: applying the Leunbach’s model

**DOI:** 10.1186/s12874-019-0768-y

**Published:** 2019-07-08

**Authors:** Núria Duran Adroher, Svend Kreiner, Carolyn Young, Roger Mills, Alan Tennant

**Affiliations:** 1 Swiss Paraplegic Research, Nottwil, Switzerland; 2 Department of Health Sciences and Health Policy, University of Lucerne, Lucerne, Switzerland; 3 Department of Biostatistics, University of Copenhagen, Copenhagen, Denmark; 4 The Walton Centre NHS Foundation Trust, Liverpool, UK; 5 University of Liverpool, Liverpool, UK

**Keywords:** Test equating, Leunbach’s model, International Classification of Functioning, Disability and Health, Rasch models, ESS, MOS, NSI, PSQI, PROMIS-SD, PROMIS-SRI

## Abstract

**Background:**

In most cases, the total scores from different instruments assessing the same construct are not directly comparable, but must be equated. In this study we aimed to illustrate a novel test equating methodology applied to sleep functions, a domain in which few score comparability studies exist.

**Methods:**

Eight scales from two cross-sectional self-report studies were considered, and one scale was common to both studies. The International Classification of Functioning, Disability and Health (ICF) was used to establish content comparability. Direct (common persons) and indirect (common item) equating was assessed by means of Leunbach’s model, which equates the scores of two scales depending on the same person parameter, taking into account several tests of fit and the Standard Error of Equating (SEE).

**Results:**

All items were linked to the body functions category b134 of the ICF, which corresponds to ‘Sleep functions’. The scales were classified into three sleep aspects: four scales were assessing mainly sleep disturbance, one quality of sleep, and three impact of sleep on daily life. Of 16 direct equated pairs, 15 could be equated according to Leunbach’s model, and of 12 indirect equated pairs, 8 could be equated. Raw score conversion tables between each of these 23 equated pairs are provided. The SEE was higher for indirect than for direct equating. Pairs measuring the same sleep aspect did not show better fit indices than pairs from different aspects. The instruments mapped to a higher order concept of sleep functions.

**Conclusion:**

Leunbach’s equating model has been successfully applied to a functioning domain little explored in test equating. This novel methodology, together with the ICF, enables comparison of clinical outcomes and research results, and facilitates communication among clinicians.

**Electronic supplementary material:**

The online version of this article (10.1186/s12874-019-0768-y) contains supplementary material, which is available to authorized users.

## Background

To measure functioning, several instruments are commonly available. Typically, one clinician or researcher uses one instrument while another uses another instrument, both of which measure the same concept. However, the scores of the two instruments cannot be directly compared as they may have different operational ranges, or measure different levels of the concept, such that their total scores are on different ordinal metrics. This restricts their comparability and impedes research and communication among clinicians.

To be able to compare scores from different instruments, they must be equated. Equating can be defined as a statistical process used to adjust scores on test forms so that the scores can be used interchangeably [1]. While various equating techniques were developed during the twentieth century, it was not until the 1980s that they became popular [1]. Equating techniques include linear, equipercentile, and Modern Test Theory (MTT) methods. They all can be used to equate scores in different data collection designs, such as those in which two or more instruments are administered to the same group of persons (known as common persons design), or those in which common items are found across different studies (known as common items design).

In linear equating, the standardized deviation scores of the two forms are set to be equal by means of a linear conversion [2]. However, the formula converting scores from one form to the other may be non-linear. Equipercentile equating admits non-linear relationships —it identifies scores on one form that have the same percentile ranks as scores on the second form— but it assumes that the scores are continuous when they are usually discrete. Although the data can be made continuous [3, 4], equipercentile relies on observed scores. MTT methods —including Item Response Theory (IRT) [5] and Rasch Measurement Theory (RMT) [6]— assume that a common latent variable lies behind responses to the items of the instruments. MTT refers to the outcomes of the latent variable as person parameters and regards an estimate of the person parameter as a measure of the respondent’s ability or trait level. IRT and RMT share a number of assumptions, including: unidimensionality, monotonicity of item characteristic functions, local independence, and no Differential Item Functioning (DIF) [7]. Testing these assumptions adds strength to the equating process, because it is possible to test that the scores of the two instruments to be equated actually measure the same construct. This test adds evidence to one of the requirements of test equating, namely construct equivalence [8].

In IRT models, the person estimate is a complex function of patterns of item responses. Compared to this, the situation is much simpler and better suited for test equating in RMT because there is a one-to-one mapping of raw scores to the estimate of the person parameter, due to the statistical sufficiency of the raw score in RMT from which follows conditional inference where assumptions about person distributions and sampling are not required, and specific objective measurement [6]. Hence, IRT and RMT are different paradigms within MTT [9].

MTT methods have been applied to create a common metric in health domains such as depression [10–12], anxiety [13], pain [14], or physical functioning [15, 16]. Furthermore, Andrich [17] presented an application of the polytomous Rasch model in equating two instruments intended to assess the same trait treating the total scores of two instruments as partial credit items from a test with two items. This approach has been employed in the health literature [18] together with the International Classification of Functioning, Disability and Health (ICF) [19] for conceptual matching. The polytomous Rasch models used in these studies are formally the same as the model described by Leunbach [20] used in this paper.

Gustav Leunbach developed the model in 1976 to assess whether two instruments measure the same trait, relating their total scores to a common scale [20]. This model is supported by a sound statistical theory on conditional inference, as well as the property of raw score sufficiency [21]. The Rasch model also possesses these properties. Although Leunbach’s model seems promising in test equating, it has rarely been applied, probably because it has gone unnoticed by the scientific community. Andrich [17] acknowledged that the model he uses came from Leunbach’s report [20], but apart from this, Leunbach’s report has rarely been cited and, as far as we are concerned, the model has not been implemented in any software until recently. Hence, it can be considered a ‘novel’ methodology which we wanted to rediscover by applying it to a functioning domain. We considered sleep functions as a case in point because it has been little explored in the field of equating.

Thus, the objective of our article is to illustrate an application of a novel methodology for equating functioning scales. Specifically, we aim (1) to rediscover Leunbach’s model and its properties, (2) to show how to interpret tests of fit and precision to decide on the adequateness of the equating, and (3) to apply the model to a domain little explored in the field of equating in health.

## Methods

### Sample and instruments

Secondary data were analysed from two cross-sectional self-report studies: Trajectories of Outcome in Neurological Conditions (TONiC), and Patient-Reported Outcomes Measurement Information System (PROMIS). These studies were chosen because they were available at the time when the current project was designed and they were suitable for secondary analyses.

The TONiC study (https://tonic.thewaltoncentre.nhs.uk/) examines the factors that influence quality of life in patients with neurological conditions. The sample for the current study consists of a cohort of patients with clinical definite Multiple Sclerosis from consecutive individual outpatient attendances in three neuroscience centres in the UK. The data were collected over the first 12 months of study recruitment, and the participants received a questionnaire pack including sleep instruments. The study had approval from the local research ethics committees. All subjects received written information on the study and gave written informed consent prior to participation [22].

The PROMIS initiative (http://www.healthmeasures.net/explore-measurement-systems/promis) aims to build item pools and develop core questionnaires that measure key health outcome domains including sleep [23]. The sample for the current study consists of an internet (YouGov Polimetrix, https://today.yougov.com/solutions/overview) sample and a clinical sample [24]. The latter included patients recruited from sleep medicine, general medicine, and psychiatric clinics at the University of Pittsburgh Medical Center.

The Epworth Sleepiness Scale (ESS) [25] was common to both studies. The Medical Outcomes Study (MOS) [26], and the three subscales of the Neurological Sleep Index (NSI) [22]—Diurnal sleepiness, Non-restorative nocturnal sleep, and Fragmented nocturnal sleep— were only present in TONiC. The Pittsburgh Sleep Quality Index (PSQI) [27], PROMIS-Sleep Disturbance [28], and PROMIS-Sleep Related Impairment [28] were only present in PROMIS.

The ESS and the PSQI are the most widely used scales in sleep medicine. However, new generic (PROMIS) and disease-specific scales are emerging and a set of these were available from the PROMIS and TONiC studies, with the ESS as the link. Hence, we took advantage of the fact of having eight sleep instruments available and we consider that equating pairwise all of them would be of interest to researchers and clinicians. The eight sleep instruments as well as the study to which each instrument was administered are described in Table 1.

**Table 1:** Description of the instruments

Instrument	Complete name	Number of items	Item (scale) range	Availability
ESS [25]	Epworth Sleepiness Scale	8	0–3 (0–24)	TONiC and PROMIS
MOS [26]	Medical Outcomes Study	6	0–4 (0–24)	TONiC
NSID [22]	Neurological Sleep Index- Diurnal sleepiness	16	0–3 (0–48)	TONiC
NSIN [22]	Neurological Sleep Index- Non-restorative nocturnal sleep	15	0–3 (0–45)	TONiC
NSIF [22]	Neurological Sleep Index- Fragmented nocturnal sleep	4	0–3 (0–12)	TONiC
PSQI [27]	Pittsburgh Sleep Quality Index^a^	14	0–3 (0–42)	PROMIS
PSD [28]	PROMIS-SD (Sleep Disturbance)	27	0–4 (0–108)	PROMIS
PSRI [28]	PROMIS-SRI (Sleep Related Impairment)	16	0–4 (0–64)	PROMIS

^a^Only the categorical items of the PSQI were considered. The sum of the individual items instead of the existing algorithm was applied

### International Classification of Functioning, Disability and Health (ICF)

The ICF is an international standard offering a common language to describe functioning [19]. It is based on the integrative bio-psycho-social model of functioning, disability and health of the World Health Organization [19]. Body functions (‘b’), Body structures (‘s’), Activities and Participation (‘d’), and Environmental factors (‘e’) are classified using an alphanumeric system. Second, third, and fourth-level categories are found under each letter, so that, for example, under the two-level category b134 sleep functions, seven third-level categories exist: b1340 Amount of sleep, b1341 Onset of sleep, b1342 Maintenance of sleep, b1343 Quality of sleep, b1344 Functions involving the sleep cycle, b1348 Sleep functions, other specified, and b1349 Sleep functions, unspecified.

One of the key requirements of test equating is construct equivalence [1]. In health, when equating scales in functioning domains, it is recommended to first link the items from the different tests to the ICF so that content comparability among the scales can be established and thus satisfy the requirement of equivalent constructs. In addition, the International Standards Organization [29] has prescribed the ICF as the framework for cataloguing health in e-health informatics (the concept of health is based on the health components of the ICF). Consequently, the use of ICF codes is two-fold in the current study: (1) to ensure concept comparability, a prerequisite for test equating, and (2) to lay a marker for the future when e-health informatics will be at the forefront of data management techniques in health care.

Hence, the items from all the scales were linked to the ICF. Two researchers performed independently the linking of items to ICF categories using the latest ICF linking rules [30], and then discussed possible disagreements to come up with a final solution. As suggested by Stucki et al. [31], the ICF Core Set for sleep disorders [32] was taken into account.

### Leunbach’s model

Leunbach [20] used a Power Series Distribution depending on an underlying common latent trait to relate the total scores of two instruments to a common scale. A Power Series Distribution [33] is a discrete probability distribution over non-negative integers of the form

2.1 $$ P\left(X=x;\xi, \gamma \right)=\frac{\xi^x{\gamma}_x}{\Gamma \left(\xi, \gamma \right)},\mathrm{x}=0,1,2,\dots; \xi \ge 0;{\gamma}_x\ge 0;\kern0.5em \Gamma \left(\xi, \gamma \right)=\sum \limits_x{\xi}^x{\gamma}_x $$

where the probability of obtaining a score *x* depends on a person parameter *ξ* and several score parameters *γ*_*x*_. For each score *x*, a score parameter is estimated.

Leunbach’s model is a test equating method for two tests (A and B), hence only the total score in each of the tests, not each item response, is considered. For each total score in A, a corresponding equated total score in B is estimated.

Let *X*_1_ be a test score of A and *X*_2_ a test score of B. (*X*_1_, *X*_2_) depend on the same person parameter *ξ*, and (A, B) have maximum scores equal to (*m*_1_, *m*_2_). The two test scores are assumed to be conditionally independent given *ξ*. Under this assumption, the probability of a total score over the test scores, *r* = *X*_1_ + *X*_2_, can be computed as

2.2 $$ P\left({X}_1+{X}_2=r|\xi \right)=\sum \limits_{x=0}^{m_1}P\left({X}_1=x|\xi \right)P\left({X}_2=r-x|\xi \right) $$

Mesbah & Kreiner [34] showed that the distributions of polytomous Rasch items can be parameterized as Power Series Distributions as described in (2.1) and that the same applies for the total score over several items including the total raw score over all items. In this sense, it is correct to regard Leunbach’s model as the joint distribution of two Rasch model super items with distributions defined by:

2.3 $$ P\left({X}_i=x|\xi \right)=\frac{\xi^x{\gamma}_{ix}}{\sum_{h=0}^{m_i}{\xi}^h{\gamma}_{ih}},\kern1.25em {\gamma}_{ix}=0\kern0.5em \mathrm{for}\ x<0\kern0.5em \mathrm{and}\ x>{m}_i $$

Then, from (2.2) and (2.3) we can derive the distribution of the total score *X*_1_ + *X*_2_:

2.4 $$ P\left({X}_1+{X}_2=r\ |\xi \right)=\frac{\xi^r{\sum}_{x=0}^{m_1}{\gamma}_{1x}{\gamma}_{2,r-x}}{\left({\sum}_{h=0}^{m_1}{\xi}^h{\gamma}_{1h}\right)\left({\sum}_{h\prime =0}^{m_2}{\xi}^{h\prime }{\gamma}_{2h\prime}\right)}=\frac{\xi^r{\omega}_r}{D} $$

where

$$ {\omega}_r=\sum \limits_{x=0}^{m_1}{\gamma}_{1x}{\gamma}_{2,r-x}\kern0.3em \mathrm{and}D=\left({\sum}_{h=0}^{m_1}{\xi}^h{\gamma}_{1h}\right)\left({\sum}_{h\prime =0}^{m_2}{\xi}^{h\prime }{\gamma}_{2h\prime}\right)=\sum \limits_{r=0}^{m_1+{m}_2}{\xi}^r{\omega}_r $$.

The joint distribution of (*X*_1_, *X*_2_) is:

2.5 $$ P\left({X}_1={x}_1,{X}_2={x}_2\ |\xi \right)=\frac{\xi^{x_1}{\gamma}_{1{x}_1}}{\sum_{h=0}^{m_1}{\xi}^h{\gamma}_{1h}}\frac{\xi^{x_2}{\gamma}_{2{x}_2}}{\sum_{h\prime =0}^{m_2}{\xi}^{h\prime }{\gamma}_{2h\prime }}=\frac{\xi^r{\gamma}_{1{x}_1}{\gamma}_{2{x}_2}}{D} $$

From this it follows that the conditional probability of the responses (*x*, *r* − *x*) of a person to the two instruments, given the person’s total score *r*, is given by the ratio (2.5) and (2.4):

2.6 $$ P\left({X}_1=x,{X}_2=r-x\ |r\right)=\frac{\gamma_{1x}{\gamma}_{2,r-x}}{\upomega_r} $$

which is independent of the person parameter *ξ* so that the total score *r* is a sufficient statistic for *ξ*. It also follows (1) that the score parameters can be estimated by the same conditional maximum likelihood estimation procedures that Andersen [35] proposed for estimates of item parameters in Rasch models, that is, by methods that make no assumptions on the distribution and sampling of persons [7]; and (2) that person parameters can also be estimated by the same maximum likelihood procedures that are used to calculate maximum likelihood estimates of person parameters in Rasch models [7].

Iterative proportional fitting [36] is used to calculate the conditional maximum likelihood estimate of score parameters and Newton-Raphson [37] to calculate the maximum likelihood estimates of person parameters.

Notice that Leunbach’s model fits raw scores from the Rasch model with conditionally independent items because the raw score over all items have Power Series Distributions. In this sense, Leunbach’s approach applies automatically. However, Leunbach’s model is more general than that, because it may apply in situations where the items of the two scores do not fit the Rasch model. The only requirement is that the two raw scores fit the Leunbach’s model. Kreiner & Christensen [38] describe loglinear Rasch models where uniform local dependence is permitted, and where the raw scores do fit Leunbach’s model.

Note also that the proposed method of equating based on Leunbach’s model could be considered as an example of Non-linear IRT True score equating [8]. Considering (*X*_1_, *X*_2_) from above, Nonlinear True score equating assumes that *X*_1_ and *X*_2_ are raw scores summarizing the responses to sets of items from IRT models with a common latent variable *θ*.

In such models, true scores ${\tau}_{X_1}$ and ${\tau}_{X_2}$ are the expected outcomes given *θ*,

2.7 $$ {\tau}_{X_1}={\nu}_{x_1}\left(\theta \right)=E\left({X}_1|\theta \right)\ \mathrm{and}\ {\tau}_{X_2}={\nu}_{x_2}\left(\theta \right)=E\left({X}_2|\theta \right) $$

The functions ${\nu}_{x_1}\left(\theta \right)$ and ${\nu}_{x_2}\left(\theta \right)$ define test characteristic curves of *X*_1_ and *X*_2_. They define a monotonic but nonlinear symmetric relationship between the true scores given by

2.8 $$ {\tau}_{X_2}={\nu}_{x_2}\left({\nu}_{x_1}^{-1}\left({\tau}_{X_1}\right)\right)\mathrm{and}{\tau}_{X_1}={\nu}_{x_1}\left({\nu}_{x_2}^{-1}\left({\tau}_{X_2}\right)\right) $$

Holland and Dorans [8] suggested to replace the true scores for observed scores in (2.8) so that one has

2.9 $$ {X}_2={\nu}_{x_2}\left({\nu}_{x_1}^{-1}\left({X}_1\right)\right)\mathrm{and}\ {X}_1={\nu}_{x_1}\left({\nu}_{x_2}^{-1}\left({X}_2\right)\right) $$

The maximum likelihood estimates of the person parameters in Leunbach’s model are equal to the person parameters where the expected value of the total score is the same as the observed score and therefore defined by ${\nu}_{x_1}^{-1}\left({X}_1\right)$ and ${\nu}_{x_2}^{-1}\left({X}_2\right)$. For this reason, we may regard the observed score as an unbiased maximum likelihood estimate of the true score and, therefore, the suggestion (2.9) is justified.

**Fig. 1:**
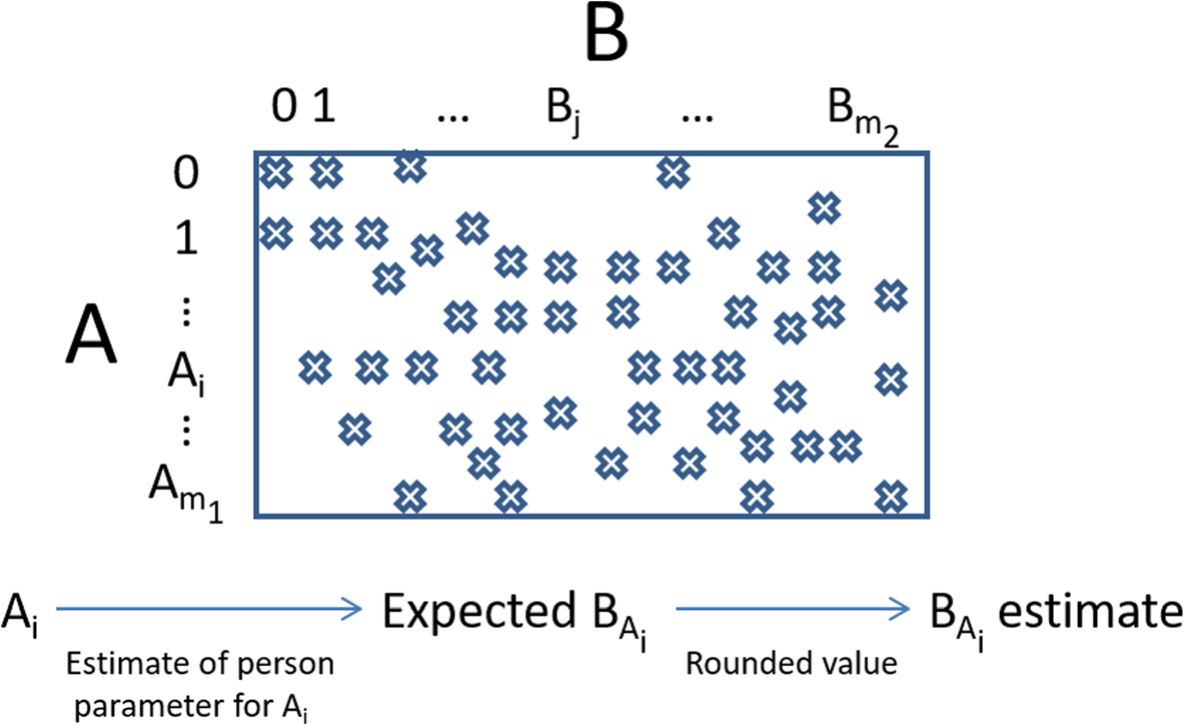
Direct equating. The crosses in the contingency table indicate a non-zero value. A_i_ is any raw score for scale A, and B_j_ is any raw score for scale B. A_m1_ is the maximum possible total score for A, and B_m2_ the maximum possible total score for B. The equating process shows that for any A_i_, an estimate for the equated value in scale B is computed given the estimate of the person parameter for A_i_

Besides, the three steps of the equating process in Leunbach’s model are the same as the steps taken in IRT true score equating, namely (1) take a score on scale A, (2) find the person parameter that corresponds to that score, and (3) find the score on scale B that corresponds to that person parameter. These steps are described in Fig. 1. More details of Leunbach’s model are given in Additional file 1.

In Leunbach’s report [20], only direct equating is addressed. In this study, we apply Leunbach’s model for both direct and indirect test equating.

#### Direct equating

For direct equating (see Fig. 1), also known as common person equating, we assume that we have two tests (A and B), and that a number of persons responds to both. This is the case, for instance, when we equate the ESS (A) to the MOS (B) from the TONiC study. In this case, the analysis by Leunbach’s model proceeds in four steps.

The first step estimates the score parameters (*γ*_*x*_) of the two tests by conditional maximum likelihood in the same way that item parameters are estimated in the Rasch model [34].

The second step tests the fit of the model to the two-way contingency table with the joint distribution of the raw scores of A and B. Since this table may be large and sparse, where we cannot rely on the asymptotic distribution of the test statistics, *p*-values are calculated by parametric bootstrapping. Bootstrapping consists of taking multiple random samples with replacement from the sample data at hand [39]. We use three tests to assess the fit of the model to the table that are similar to tests used to test for multidimensionality in Rasch models. First, (1) a conditional Likelihood Ratio Test comparing observed and expected counts given the total score of the two tests. Second, (2) a test comparing the observed correlation (Goodman and Kruskal’s Gamma [40]) of the scores to the expected value under the model. Horton et al. describe a similar test of unidimensionality for Rasch models [41]. Third, (3) by counting the number of persons with two scores that depart significantly at a 5% critical level from each other under the Leunbach’s model. Since the person parameter of Leunbach’s model can be estimated separately from the two scores, this test is similar to a t-test of unidimensionality in Rasch models comparing person estimates from different subscores [42]. The advantage of focusing on subscores instead of person parameters is that the analysis avoids the problematic assumption that person estimates are normally distributed. A chi-square *p*-value is obtained for (3) on whether the observed frequencies of persons with significant differences is larger than 5%. Following Cox and Snell (page 37) [43] we only regard *p*-values less than or equal to 0.01 as strong evidence against the fit of the model to the data. Moderate evidence provided by *p*-values less than 5% will of course occur, but will only be regarded as conclusive if more than one of the three tests are significant. However, the reader is free to draw their own conclusions concerning model fit in Table 5.

The third step equates a score on A to a score on B: Firstly, by calculating a maximum likelihood estimate of the person parameter given the A score, and secondly, by calculating the expected B score for persons with a person parameter equal to this estimate. Since the equated B score has to be an integer instead of a real number, the equated B score is defined as the rounded value of the expected B score.

The final step assesses the error of equating by bootstrapping from the observed contingency table. If the model was accepted during step two, the variation of the results of the three steps on the bootstrapped data will provide an unbiased estimate of the random error associated with the equated results. Such error is the Standard Error of Equating (SEE) [1] and is computed for each equated score. In other words, the SEE corresponds to the standard deviation of equated scores over hypothetical replications of an equating procedure in samples from a population of test takers [1]. For a score *x*_*i*_ of test A, the SEE of the equated score on test B, ${\hat{eq}}_B\left({x}_i\right)$, can be computed using the following formula

$$ se\left[{\hat{eq}}_B\left({x}_i\right)\right]=\sqrt{\mathit{\operatorname{var}}\left[{\hat{eq}}_B\left({x}_i\right)\right]}=\sqrt{\mathbf{E}{\left\{{\hat{eq}}_B\left({x}_i\right)-\mathbf{E}\left[{\hat{eq}}_B\left({x}_i\right)\right]\right\}}^{\mathbf{2}}} $$

We calculated the replications of the equating procedure in S = 1000 bootstrap samples. The SEE formula using bootstrap samples is as follows:

$$ {\hat{se}}_{boot}\left[{\hat{e}}_B\left({x}_i\right)\right]=\sqrt{\frac{\sum \limits_{\boldsymbol{s}}{\left\{{\hat{e}}_{B_s}\left({x}_i\right)-{\hat{e}}_{B.}\left({x}_i\right)\right\}}^{\mathbf{2}}}{\mathrm{S}-1}}, $$

where

$$ {\hat{e}}_{B.}\left({x}_i\right)=\frac{\sum \limits_{\boldsymbol{s}}{\hat{e}}_{B_s}\left({x}_i\right)}{S} $$

A weighted SEE mean for all the equated scores is then calculated. We calculated a weighted instead of an unweighted mean because we are summarizing errors over a large number of score groups, some of them with very few cases, and an unweighted mean would mean that the errors in the small groups would inflate the assessment of the degree of error in the population.

As explained in Table 2 and in Additional file 1, we regard a weighted SEE mean below 0.91 as acceptable.

**Table 2:** Standard Error of Equating

A	Expected B	B estimate	SEE	Relative frequency of bootstrap errors				
				−2+	−1	0	1	2+
0	0	0	0	0	0	1	0	0
1	1.9	2	0.316	0	0.05	0.9	0.05	0
12	18.3	18	0.81	0.025	0.225	0.5	0.225	0.025
20	35.4	35	0.91	0.025	0.317	0.317	0.317	0.025

This table contains artificial values of equated scores from scale A to B, with different distributions of the bootstrap errors. For each raw score of A an estimated raw score of B is obtained. The SEE (Standard Error of Equating) is computed from the second half of the table, where, for each A raw score, 1000 bootstrapped B scores are estimated in 1000 bootstrap samples. The difference (error) between the B estimate and each of the bootstrapped B scores is computed. The number of errors of 0 points (no error), 1 point below (− 1), two or more points below (− 2+), 1 point above (1), and two or more points above (2+) the B estimates are collected. Then the Relative Frequencies (RF) of these errors are presented on the table, which allow to compute the SEE. Four theoretical bootstrap error distributions are presented. The first row shows an error free distribution, where RF(0) = 1 and therefore SEE = 0. The second row shows a plausible distribution, where RF(0) = 0.9, RF(− 1) = RF(1) = 0.05, and it follows that SEE= $\sqrt{0.05+0.05}$ =0.316. The third row shows an acceptable distribution, where RF(0) = 0.5, RF(− 1) = RF(1) = 0.225, RF(− 2+) = RF(2+) = 0.025, and it follows that SEE= $\sqrt{2\ast 0.225+2\ast 4\ast 0.025}=\sqrt{0.65}$ =0.81. The fourth row shows the worst case that could be regarded as acceptable, where RF(0) = RF(− 1) = RF(1) = 0.317, RF(− 2) = RF(2) = 0.025, and it follows that SEE is 0.91. We therefore consider a weighted SEE mean below 0.91 as acceptable

*Abbreviations: SEE* Standard Error of Equating

#### Indirect equating

For indirect equating, also known as common item equating, imagine that we have three tests (A, B, and C); one sample of persons responds to A and B, and another sample responds to A and C. Equating from B to C can be indirectly done via A, which is the ‘common item’ (or common scale) enabling the equating. This is the case, for instance, when we equate the MOS (B)—available only in the TONiC sample, to the PSQI (C)—available only in the PROMIS sample, via the common scale ESS (A)—available in both TONiC and PROMIS samples.

The scale A should not work differently for the two samples of persons. Therefore, Differential Item Functioning (DIF) [44] for sample was assessed in each indirect equating triplet A, B, C.

**Fig. 2:**
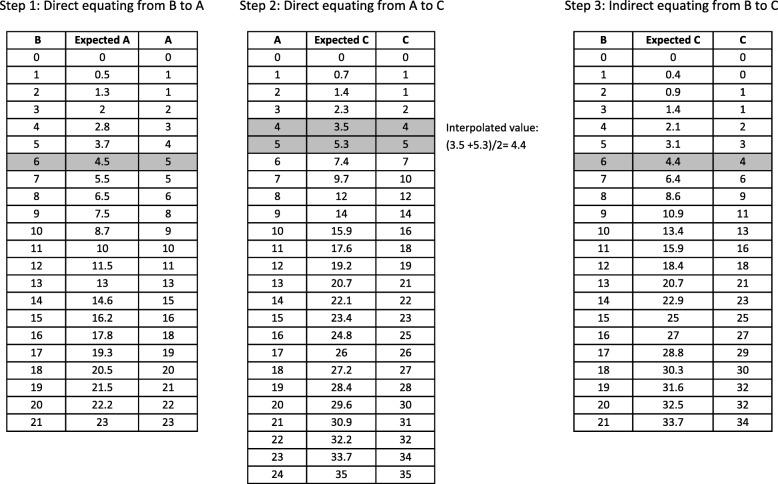
Indirect equating. This figure shows the three-step procedure to equate test B to test C indirectly via test A. The direct equating of B to A and the direct equating of A to C are the two previous steps needed to conduct the indirect equating from B to C

Indirect equating from B to C is a three-step procedure. In the first step, direct equating of B to A is performed. In the second step, direct equating of A to C is performed. Then, the results of the previous steps are used to establish a correspondence of scores from B to C (i.e., to perform indirect equating). For example, as shown in Fig. 2, imagine that we want to know the score of C that corresponds to a score of 6 of B. We first have to find in step 1 the expected score in A of 6, which is 4.5. Then in step 2 we see that the expected scores for A = 4 and A = 5 are 3.5 and 5.3, respectively. Hence, the expected C score lies between 3.5 and 5.3, and by interpolating we find that it is (3.5 + 5.3)/2 = 4.4, which corresponds to a rounded integer of 4.

The tests of fit (second step in section Direct equating) are not available for indirect equating because to evaluate misfit the contingency table shown in Fig. 1 is needed, and it cannot be obtained if different sets of persons have responded to the tests. Nevertheless, in the first two steps of indirect equating from B to C via A, it is tested whether B and A measure the same construct, and whether A and C measure the same construct. If both tests accept the hypotheses, it follows logically that B and C must measure the same construct. On the other hand, the SEE of the indirect equating from B to C can be estimated by bootstrapping in exactly the same way as for direct equating. In addition, Additional file 1: Table S20 provides an example where the ESS and the MOS are equated directly and indirectly via the NSID, and the score correspondences in both cases are very similar.

### Software

Direct and indirect equating pairs among the eight sleep instruments were assessed by the Leunbach’s model implemented in DIGRAM [45], which is free and can be downloaded from http://staff.pubhealth.ku.dk/~skm/skm/. Additional file 1 shows how to perform Test Equating with DIGRAM. DIF was assessed with RUMM2030 [42]. The statistical test used for detecting DIF in RUMM2030 is a two-way Analysis of Variance (ANOVA) [46] of the person-item deviation residuals with person factors (i.e. sample) and class intervals (i.e., strata along the trait) as factors.

## Results

### Sample

The TONiC sample consisted of 722 multiple sclerosis patients, and the PROMIS sample of 2252 participants recruited from the internet and from clinics. Of the 1993 participants from the PROMIS internet sample, 1259 reported no sleep problems and 734 reported sleep problems. The clinical sample consisted of 259 adults from clinics at the University of Pittsburgh Medical Center. Table 3 shows the distribution of sex and age in the TONiC and PROMIS samples, as well as globally.

**Table 3:** Distribution of sex and age by sample

Variable	TONiC *n* = 722	PROMIS *n* = 2252	Total *n* = 2974
	n (%)	n (%)	n (%)
Sex			
Male	197 (27.3)	1167 (51.8)	1364 (45.9)
Female	519 (71.9)	1085 (48.2)	1604 (53.9)
Missing	6 (0.8)	0 (0)	6 (0.2)
Age			
< =40	160 (22.2)	575 (25.5)	735 (24.7)
41–50	221 (30.6)	507 (22.5)	728 (24.5)
51–59	183 (25.3)	494 (21.9)	677 (22.8)
> =60	131 (18.1)	676 (30)	807 (27.1)
Missing	27 (3.7)	0 (0)	27 (0.9)

### ICF

The 106 items of the 8 instruments were linked to the second level ICF category b134 sleep functions. Some were also linked to a third level sleep category (b1340 Amount of sleep, b1341 Onset of sleep, b1342 Maintenance of sleep, b1343 Quality of sleep). The b categories in the brief ICF Core Set for sleep disorders—b134 Sleep functions, b130 Energy and drive functions, b140 Attention functions, b110 Consciousness functions, and b440 Respiration functions, were found in our linking; while b134 was the primary concept, the rest were secondary concepts. Three of the four Core Set d categories—d475 Driving, d240 Handling stress and other psychological demands, d230 Carrying out daily routine, were also found as secondary concepts. More b, d, and e categories were identified as secondary concepts, too. All these secondary categories are the contextual parameters for items in the sleep instruments.

Five main sleep aspects —Sleep disturbance (b1341, b1342), Quality of Sleep (b1343), Amount of sleep (b1340), Impact of sleep on daily life (b134), Facilitators/barriers of sleep (b134), to which each item could belong to, were derived. Table 4 shows the number of items per instrument belonging to a sleep aspect.

**Table 4:** Number of items belonging to a sleep aspect per instrument

Instrument	Sleep disturbance	Quality of sleep	Amount of sleep	Impact of sleep on daily life	Facilitators/ barriers of sleep
ESS				***8***	
MOS	***3***	2	1		
NSID				***16***	
NSIN	4	***9***	2		
NSIF	***3***	1			
PSQI	***9***	1		2	2
PSD	***18***	8	1		
PSRI		4		***12***	
Total number of instruments	4	1		3	

The numbers in bold-italics refer to the most prevalent aspect

MOS, NSIF, PSQI, PSD were assessing mainly sleep disturbance, NSIN quality of sleep, and ESS, NSID, PSRI impact of sleep on daily life. ESS and NSID were the sole instruments with all the items pointing to one sleep aspect. NSIF and PSRI involved two aspects, MOS, NSIN, and PSD three, and PSQI four.

The two PSQI items belonging to Facilitators/barriers of sleep (*How often have you taken medicine to help you sleep (prescribed or ‘over the counter’)? / Do you have a bed partner or roommate?*) were not considered in the summated score. They are Environmental factors in ICF nomenclature, and thus cannot be summated with the other items. The PSQI ended up with 12 items, and with a score range of 0–36.

### Leunbach’s model

For each pairwise direct equating, DIGRAM uses the estimates of the score parameters to calculate the expected counts under the Leunbach’s model and to test whether the model fits the data. Three test of fit are available (Likelihood ratio test, Gamma coefficient, and the Number and percentage of persons with significant differences between measurements). A bootstrap *p*-value is provided for the first and second tests, and an asymptotic chi square *p*-value is obtained for the third. These *p*-values are presented in Table 5 (columns 2–4) for each directly equated pair, highlighting the *p*-values with a significant level below 0.01. The equating of ESS-PSD, ESS-PSRI, and PSD-PSRI are presented as a percentage of persons with significant differences between measurements. ESS-PSD presented also a significant gamma coefficient, so there is evidence from two tests that ESS and PSD measure different constructs; equating these two tests or using them for indirect equating was therefore not recommended. MOS-NSIF and NSIN-NSIF presented a significant Likelihood Ratio Test.

**Table 6:** Indirect equating

Equated pair	Bootstrap distribution of SEE			
	Min	Median	Max	Weighted Mean
MOS^b^-PSQI^b^ PSQI^b^-MOS^b^	0	0.73	1.37	0.66
	0	0.48	1.17	0.36
MOS^b^-PSRI^a^ PSRI^a^-MOS^b^	0	1.54	2.25	**1.23**
	0	0.45	1.35	0.38
NSID^a^-PSQI^b^ PSQI^b^-NSID^a^	0	0.61	3.27	0.63
	0.06	0.71	1.96	0.77
NSID^a^-PSRI^a^ PSRI^a^-NSID^a^	0	1.23	4.99	**1.19**
	0	0.61	2.27	0.75
NSIN^c^-PSQI^b^ PSQI^b^-NSIN^c^	0	0.64	1.41	0.69
	0.06	0.79	2.17	**0.92**
NSIN^c^-PSRI^a^ PSRI^a^-NSIN^c^	0	1.14	2.28	**1.33**
	0.03	0.78	2.35	**0.94**
NSIF^b^-PSQI^b^ PSQI^b^-NSIF^b^	0	0.7	1.49	0.81
	0	0.34	0.56	0.28
NSIF^b^-PSRI^a^ PSRI^a^-NSIF^b^	0	1.17	2.27	**1.4**
	0	0.33	0.61	0.27
**Ideal value**				**<0.91**

Pairs involving PSD are excluded because they involve the equating ESS-PSD or PSD-ESS, which is not recommended

Average weighted means above 0.91 are in bold

*Abbreviations*: *SEE* standard error of equating

^a^Most prevalent aspect is Sleep disturbance

^b^Most prevalent aspect is Impact of sleep on daily life

^c^Most prevalent aspect is Quality of sleep

To assess the precision of the equating results, for each equated score in each equated pair, bootstrap samples were generated in order to compute the standard deviation of the equated scores over replications, namely the SEE. The distribution of the SEE among the equated scores for each equated pair is presented in the last four columns of Table 5. The most relevant value is the weighted mean, and values above 0.91 are highlighted. The minimum SEE values were practically 0 for all the pairs, and the maximum oscillated between 0.5 and 3.55. The weighted SEE mean is below 1 in all the pairs except ESS-PSD.

The indirect equated pairs (via ESS) excluding the PSD ones (which involved ESS-PSD) were first tested for DIF by sample. ESS showed DIF only for NSIF-PSQI, and the marginal value was considered not to be substantial enough to prevent the equating. Then we assessed the tests of fit in the first two direct equating steps: if these were acceptable, the fit of the indirect equating was also acceptable. The fit was acceptable for all the pairs except the ones involving ESS-PSD. Regarding the SEE, bootstrap samples were generated and evaluated. Table 6 shows the distribution of the SEE for each pairwise indirect equating excluding PSD pairs. The SEE values were higher than for direct equating, oscillating the maximum between 0.56 and 4.99, and the highest weighted mean value was 1.4. The pairs involving PSRI presented a weighted mean above 1.

**Table 5:** Direct equating

Equated pair	Likelihood Ratio Test	Gamma coefficient	Number (%,CI, and *p*-value^*^) of persons with significant differences between measurements	Bootstrap distribution of SEE			
				Min	Median	Max	Weighted Mean
ESS^a^-MOS^b^ MOS^b^-ESS^a^	*P* = 0.604	*P* = 0.606	31 (4.6%) [3.0, 6.2] *P* = 0.6515	0	0.48	0.75	0.39
				0	0.5	0.74	0.46
ESS^a^-NSID^a^ NSID^a^-ESS^a^	*P* = 0.140	*P* = 0.988	33 (5.3%) [3.5, 7] *P* = 0.7481	0.06	0.66	2.14	0.80
				0	0.46	2.18	0.38
ESS^a^-NSIN^c^ NSIN^c^-ESS^a^	*P* = 0.112	*P* = 0.930	42 (6.7%) [4.7, 8.6] *P* = 0.0563	0.06	0.66	2.37	**0.93**
				0	0.47	0.82	0.43
ESS^a^-NSIF^b^ NSIF^b^-ESS^a^	*P* = 0.576	*P* = 0.658	32 (4.8%) [3.2, 6.4] *P* = 0.8172	0	0.3	0.5	0.24
				0	0.48	0.85	0.44
ESS^a^-PSQI^b^ PSQI^b^-ESS^a^	*P* = 0.027	*P* = 0.492	133 (6%) [5, 7] *P* = 0.0362	0	0.5	1.23	0.36
				0	0.43	1.02	0.23
ESS^a^-PSD^b^ PSD^b^-ESS^a^	*P* = 0.134	*P* = 0.002^d^	146 (6.5%) [5.5, 7.6] *P* = 0.0009^d^	0	1.26	2.9	**1.35**
				0	0.32	2.08	0.21
ESS^a^-PSRI^a^ PSRI^a^-ESS^a^	*P* = 0.064	*P* = 0.840	160 (7.1%) [6.1, 8.2] *P* = 0.0000^d^	0	0.89	2.02	0.58
				0	0.33	1.46	0.21
MOS^b^-NSID^a^ NSID^a^-MOS^b^	*P* = 0.020	*P* = 0.999	38 (6%) [4.2, 7.9] *P* = 0.2428	0.03	0.78	1.67	0.81
				0	0.44	0.78	0.38
MOS^b^-NSIN^c^ NSIN^c^-MOS^b^	*P* = 0.168	*P* = 0.883	34 (5.3%) [3.6, 7.0] *P* = 0.7238	0	0.55	2.25	0.71
				0	0.4	0.54	0.35
MOS^b^-NSIF^b^ NSIF^b^-MOS^b^	*P* = 0.006^d^	*P* = 0.994	30 (4.4%) [2.9, 6] *P* = 0.5205	0	0.2	0.5	0.23
				0	0.31	0.53	0.29
NSID-^a^NSIN^c^ NSIN^c^-NSID^a^	*P* = 0.060	*P* = 0.990	42 (6.9%) [4.9, 8.9] *P* = 0.0336	0	0.6	1.55	0.58
				0.06	0.71	1.48	0.60
NSID^a^-NSIF^b^ NSIF^b^-NSID^a^	*P* = 0.200	*P* = 1.000	34 (5.4%) [3.6,7.1] *P* = 0.682	0	0.38	0.74	0.27
				0.07	1.04	1.64	**0.92**
NSIN^c^-NSIF^b^ NSIF^b^-NSIN^c^	*P* = 0.001^d^	*P* = 1.000	33 (5.1%) [3.4,6.9] *P* = 0.8633	0	0.38	0.65	0.27
				0	0.78	2.14	0.85
PSQI^b^-PSD^b^ PSD^b^-PSQI^b^	*P* = 0.300	*P* = 0.177	127 (5.7%) [4.7,6.7] *P* = 0.1294	0	1.05	1.72	0.70
				0	0.37	1.68	0.26
PSQI^b^-PSRI^a^ PSRI^a^-PSQI^b^	*P* = 0.234	*P* = 0.018	131 (5.9%) [4.9, 6.8] *P* = 0.0603	0	0.70	2.04	0.48
				0	0.42	1.67	0.26
PSD^b^-PSRI^a^ PSRI^a^-PSD^b^	*P* = 0.085	P = 1.000	177 (7.9%) [6.8,9.0] P = 0.000^d^	0	0.5	2.43	0.42
				0	0.87	3.55	0.68
**Ideal values**	**> 0.01**	**> 0.01**	**Lower Confidence** **Interval < 5%**			**<0.91**	

Average weighted means above 0.91 are in bold

*Abbreviations*: *P p*-value, *SEE* Standard error of Equating

^a^Most prevalent aspect is Sleep disturbance

^b^Most prevalent aspect is Impact of sleep on daily life

^c^Most prevalent aspect is Quality of sleep

^d^Significant at the 1% level

^*^The *p*-value of a test that the observed frequencies of persons with significant differences is larger than 5%

Additional file 1 contains detailed results of the direct equating of ESS and MOS and of the indirect equating of MOS and PSQI via ESS.

Tables 5 and 6 show that pairs belonging to the same aspect did not necessarily have better fit indices and precision than pairs from different aspects. For example, MOS-ESS (different aspects) shows better fit values than PSQI-PSD (same aspect). While MOS-PSQI (same aspect) shows better SEE values than MOS-PSRI (different aspects), NSID-PSQI (different aspects) shows better SEE values than NSID-PSRI (same aspect). Also, both tables show that the SEE is lower when we equate the large scale (in terms of scale range) to the small one than vice versa. For example, the SEE for ESS-NSID (small to large) is 0.80 while NSID-ESS (large to small) is 0.38.

Out of the 28 possible pairs, 23 could be equated. The exchange tables for these 23 equated pairs can be found in Additional file 2.

## Discussion

In this study we described a novel methodology for equating functioning scales and we applied it to a domain little explored in the field of equating, sleep functions. Leunbach’s model equates the scores of two scales considering that they depend on the same person parameter. It has been shown how to take into account the three tests of fit, as well as the SEE, to decide on the adequateness of the equating.

In our case in point, 23 out of the 28 possible pairs among 8 instruments could be equated according to the model. The reason why the Gamma coefficient, and the counting of the number of persons with two scores that depart significantly at a 5% critical level from each other under the model are significant for equating ESS-PSD, could be due to a type 1 error. In addition, the scale range difference between ESS and PSD, 84, is the highest among all the direct equated pairs. The higher this difference is, the more problematic is the equating.

Issues remain for ESS-PSRI, PSD-PSRI, MOS-NSIF, and NSIN-NSIF. Their misfit may be due to local dependence between scores and/or because the latent trait assumed by the Leunbach’s model to lie behind the scores is measured on logit scales with different units [47]. While equating the ESS with the PSD should be avoided, the scores of ESS-PSRI, PSD-PSRI, MOS-NSIF, and NSIN-NSIF could be equated. The indirect equating was free of DIF for sample with one exception showing marginal DIF without impeding the equating.

The SEE for indirect equating was larger than for direct equating because the former uses results from two sets of direct equating estimates, both of which have error. Indirect equating is, therefore, less robust than direct. We also observed that there is less precision in terms of SEE when we equate the small scale (having a lower score range) to the large one (having a bigger score range) than vice versa. This makes sense because when going from small to large, for each score there is a wider range of options of scores to be equated.

As explained in the Methods section, when equating scales in functioning domains, linking the items to the ICF enables to establish content comparability among the scales and thus satisfy the requirement of construct equivalence [1]. In our case in point, the instruments were classified into three sleep aspects: sleep disturbance, quality of sleep, and impact of sleep on daily life. Given that the pairs belonging to the same aspect did not necessarily present better fit indices than pairs from different aspects, it seems that the instruments map to a higher order concept of sleep functions (b134). Moreover, as only 2 (ESS and NSID) of the 8 instruments were measuring one sole aspect, different aspects of sleep are already considered in the existing instruments. ESS and NSID are then more limited than the remaining instruments, which are more content valid. Hence, the linking process helped also in the interpretation of the results.

Sleep scales have been previously linked to the ICF [48], and the ICF has also been used to compare the content of health status measures, where the b134 sleep functions category appears [49–51], or where the content relates to sleep medicine practice and research [52, 53]. The PSQI has also been linked to the ICF together with instruments from other health domains [54]. Problems in functioning of people with sleep disorders have also been identified via the ICF [55–57]. However, we are unaware of any study that uses the ICF beyond the content comparability to formally equate sleep scales.

Leunbach’s model, developed by Gustav Leunbach in 1976, has been rarely applied despite its desirable properties of raw score sufficiency, sound statistical theory on conditional tests, and the similarity with Rasch models for measurement. This similarity should not be surprising; Leunbach collaborated with Rasch for many years (Leunbach translated —or, according to Rasch, transformed— Rasch’s 1960 book [6] from Danish into English; see page ix of the book [6]) and it is not an unreasonable conjecture that the idea of using power series distributions for measurement models came from Rasch himself. The similarity between the power series distribution and the distribution of test scores in Rasch’s multiplicative Poisson model and the distribution raw score in the Rasch model for item analysis (see formula (5.5) in Chapter X of the Rasch’s book [6]) is also an indicator of the inspiration for Leunbach’s model.

A limitation of this study, considering the current implementation of the Leunbach’s model in DIGRAM, is that only the raw scores taken by the sample appear in the equating table. In our case in point, this is the case of MOS, which theoretical range is 0–24 but only the range 0–21 is equated, because the raw scores 22–24 were not taken. This problem could be solved by interpolation, and we are currently working on how to implement it in DIGRAM with the aim that the next version of DIGRAM will incorporate it. Another limitation is that the ESS, the common scale used for indirect equating, assesses only one sleep aspect (impact of sleep on daily life), and therefore the indirect equating is not optimal. Nevertheless, we have shown that it is possible to equate several sleep scales using the Leunbach’s model. The exchange of scores between pairs of sleep instruments available in Additional file 2 will facilitate the comparison of clinical outcomes and research results. Any clinician or researcher can continue using the sleep scale they feel more comfortable with and look for the correspondence of each raw score to any other sleep scale.

In this study we applied a particular test equating methodology to two specific datasets. Hence, the results obtained are not generalizable. Although the main focus of this study was not to provide generalizable findings, but to illustrate the application of a novel test equating method, it would be interesting to carry out in future studies simulations on different testing conditions to assess the robustness of Leunbach’s model. Another future research study could compare Leunbach’s model to other equating methods. DIGRAM also provides equating results from the equipercentile method, and Additional file 1 includes the equipercentile results from ESS and MOS equating.

In conclusion, we illustrated how to apply a novel test equating methodology implemented (partly during the current study) in the DIGRAM software which is free and is easy to use. We encourage its use in future applications.

## Additional files

Additional file 1: Direct and indirect test equating in DIGRAM 4.06. (PDF 699 kb)

Additional file 2: Raw score conversion tables among sleep instruments. (XLSX 28 kb)

## Data Availability

The datasets used and/or analysed during the current study are available from the corresponding author on reasonable request.

## Abbreviations

DIF: Differential Item Functioning
ESS: Epworth Sleepiness Scale
ICF: International Classification of Functioning, Disability and Health
IRT: Item Response Theory
MOS: Medical Outcomes Study
MTT: Modern Test Theory
NSI: Neurological Sleep Index
NSID: NSI-Diurnal sleepiness
NSIF: NSI-Fragmented nocturnal sleep
NSIN: NSI-Non-restorative nocturnal sleep
PROMIS: Patient-Reported Outcomes Measurement Information System
PSD: PROMIS-Sleep Disturbance
PSQI: Pittsburgh Sleep Quality Index
PSRI: PROMIS-Sleep Related Impairment
RMT: Rasch Measurement Theory
SEE: Standard Error of Equating
TONiC: Trajectories of Outcome in Neurological Conditions
