# Supplementary material for: Test equating sleep scales: applying the Leunbach’s model
Source: BMC Med Res Methodol. 2019 Jul 8;19:141. doi: 10.1186/s12874-019-0768-y (PMC6613254; doi:10.1186/s12874-019-0768-y)
Supplement: Supplementary file 1 — Direct and indirect test equating in DIGRAM 4.06. (PDF 699 kb) [file 12874_2019_768_MOESM1_ESM.pdf]

Direct and indirect test equating in DIGRAM 4.06

Svend Kreiner and Núria Duran Adroher, February 2019

Table of contents.

1 Introduction

2 The Leunbach model

3 Test equating by the Leunbach model

4 Data

5 Direct test equating in DIGRAM

6 Indirect test equating in DIGRAM

7 References

The following pages are meant as an informal user guide to test equating by the Leunbach model in DIGRAM (Kreiner and Nielsen 2013). They explain and illustrate the Leunbach model and provide information on the analysis, but assume that the reader is familiar with DIGRAM.

1 Introduction

Assume that you are interested in equating two or three different test scores, Test1, Test2, and Test3, measuring the same trait. Depending on the data collection design, direct or indirect test equating has to be performed.

Direct test equating between two test scores of Test1 and Test2 assumes that you have observations of the test scores for the same set of persons.

If you have observations of Test1 and Test2 for one set of persons and observations of Test1 and Test3 for a different set of persons, but no persons with joint observations of Test2 and Test3, it is possible to establish indirect equating from Test2 to Test3 by applications of results from direct equating of Test1 and Test2 and of Test1 and Test3.

DIGRAM uses Leunbach (1976) unidimensional power series model for both direct and indirect equating.

In both cases, test equating has to address the same issues.

First, to test that the unidimensional Leunbach model fits the data. During indirect equating where we assume that there are no persons with joint observations of Test2 and Test3, the test of fit consists of tests of fit to the Leunbach model to Test1 & Test2 and Test1 & Test 3. If these tests accept that Test1 and Test2 measure the same construct ant that Test1 and Test 3 also measure the same construct it follows logically that the same applies to Test 2 and Test 3.

Second, to estimate a score on the second test corresponding to a score on the first.

Third, to assess the error of the estimates of the equated scores.

The following pages define the Leunbach model, explain how test equating by the Leunbach model relates to other procedures and methods for test equating and finally show how to perform test equating in DIGRAM.

2 The Leunbach model

Leunbach (1976) used so-called power series distributions (PSD) (Noack 1950) to describe the relationship between test scores depending on a common latent trait.

Let X be a random variable with a range consisting of a set of non-negative integers. We say that X belongs to the family of PSD distributions and that the distribution of X is a PSD(ξ,γ) distribution if

the probabilities depend on an effect parameter ξ and a set of non-negative score parameters γ = $\{\gamma_0, \gamma_1, \gamma_2, \ldots\}$ in the following way:

$$\text{Prob}(X = x; \xi, \gamma) = \frac{\xi^x \gamma_x}{\Gamma(\xi, \gamma)} \tag{1}$$

In Formula (1), $\Gamma(\xi,\gamma) = \sum_x \xi^x \gamma_x$. A score parameter γ_x is equal to zero if x is outside the range of X. Probabilities depend on the product of the effect parameter ξ and the score parameters. For this reason, restrictions are required to make parameters identifiable. Because the Leunbach model assumes that the range of X includes zero, the Leunbach model assumes that $\gamma_0 = 1$.

Let Test1 and Test2 be two tests and that v is a person being administered both tests. Leunbach's model makes the following assumptions relating to two test scores, X_v from Test1 and Y_v from Test2, v = 1,..,n

1) X_v and Y_v depend on the outcome θ_v of a latent trait variable Θ_v.
2) X_v and Y_v are conditionally independent given Θ_v.
3) The conditional distribution of X_v and Y_v given $\Theta_v = \theta$ are respectively PSD($e^\theta, \gamma^{(X)}$) and PSD($e^\theta, \gamma^{(Y)}$) where $\gamma^{(X)} = \{\gamma_0^{(X)}, \gamma_1^{(X)}, \ldots \gamma_x^{(X)}, \ldots\}$ and $\gamma^{(Y)} = \{\gamma_0^{(Y)}, \gamma_1^{(Y)}, \ldots \gamma_y^{(Y)}, \ldots\}$ are the score parameters of X and Y.

During the discussion of the analysis and the presentation of the results we sometimes use the logarithm, $\theta = \ln(\xi)$ as an effect parameter because users of IRT models and Rasch models are used to measure latent trait on interval scales. During analyses of the relationships between the scores, it has no effect at all whether we use one or the other parameterization.

Given these assumptions, the joint conditional distribution of X_v and Y_v given Θ_v is shown in Formula (2), where to simplify things we ignore the indices referring to a specific person

$$\text{Prob}(X = x, Y = y \mid \theta; \gamma^{(X)}, \gamma^{(Y)}) = \frac{e^{(x+y)\theta} \gamma_x^{(X)} \gamma_y^{(Y)}}{\Gamma(e^\theta, \gamma^{(X)}) \Gamma(e^\theta, \gamma^{(Y)})} \tag{2}$$

Formula (2) shows that T = X+Y is sufficient for θ and therefore that the *conditional* distribution of (X,Y) *given* T does not depend on θ. Formula (3) shows the distribution of T and Formula (4) the *conditional* distribution of (X,Y) *given* T.

$$\text{Prob}(T = t \mid \theta; \gamma^{(X)}, \gamma(^{(Y)}) = \sum_{(x,y):x+y=t} \frac{e^{(x+y)\theta} \gamma_x^{(X)} \gamma_y^{(Y)}}{\Gamma(e^\theta, \gamma^{(X)}) \Gamma(e^\theta, \gamma^{(Y)})} = \sum_t \frac{e^{t\theta} \gamma_t^{(T)}}{\Gamma(e^\theta, \gamma^{(T)})} \tag{3}$$

where $\gamma_t^{(T)} = \sum_{(x,y):x+y=t} \gamma_x^{(X)} \gamma_y^{(Y)}$.

Finally, dividing (2) by (3) gives us the conditional distribution of (X,Y) given T which is independent of θ.

$$\mathrm{Prob}(X=x, Y=y \mid T=t; \gamma^{(X)}, \gamma^{(Y)}) = \frac{e^{(x+y)\theta}\,\gamma_x^{(X)}\gamma_y^{(Y)}}{e^{t\theta}\,\gamma_t^{(T)}} = \frac{\gamma_x^{(X)}\gamma_y^{(Y)}}{\gamma_t^{(T)}} \tag{4}$$

Formulas (2) - (4) posit the Leunbach model in the family of measurement models defined by Rasch (1960), where conditional inference separates inference on instrument parameters from person parameters and vice versa. The connection between Rasch's measurement model and Leunbach's model should also be apparent for the following reasons. First, because Rasch's multiplicative Poisson model is a special case of Leunbach's model where the score parameters are functions of a single instrument parameter and second, because the distribution of the total score over a set of items from Rasch's model for dichotomous items is a PSD distribution, where score parameters are defined by the symmetrical polynomials over item parameters.

We refer to Kreiner and Mesbah (2013) for additional amplifications of the relationship between the PSD distributions and Rasch models. For now, it suffices to point out that

1) The score parameters of X and Y can be estimated by the same kind of conditional maximum likelihood methods that Andersen (1970) proposed for estimates of item parameters in Rasch models. That is, by methods that make no assumptions on the distribution and sampling of persons.
2) The ML estimates of person parameters can be calculated by the same procedures that are used to calculate ML estimates of person parameters in Rasch models.

Leunbach's model can provide estimates of the person parameters based on X, Y, and T.

Let $\hat{\gamma}^{(X)}$ and $\hat{\gamma}^{(Y)}$ be the CML estimates of the score parameters of X and Y and let $\hat{\gamma}^{(T)}$ be estimates of the score parameters of T defined by $\hat{\gamma}_t^{(T)} = \sum_{(x,y):x+y=t} \hat{\gamma}_x^{(X)}\hat{\gamma}_y^{(Y)}$. To calculate maximum likelihood estimates of ξ and θ=ln(ξ) based on X, Y and T we have to solve the following three equations, where $\hat{\xi}_x^{(X)}$ is the ML estimate given X=x, $\hat{\xi}_y^{(Y)}$ is the ML estimate given Y=y, $\hat{\xi}_t^{(T)}$ is the ML estimate given T=t:

$$x = E\left(X \mid \hat{\xi}_x^{(X)}\right) = \sum\nolimits_s s \frac{\left(\hat{\xi}_x^{(X)}\right)^s \hat{\gamma}_s^{(X)}}{\Gamma\left(\hat{\xi}_x^{(X)}, \hat{\gamma}^{(X)}\right)} \tag{5}$$

$$y = E\left(Y \mid \hat{\xi}_y^{(Y)}\right) = \sum\nolimits_s s \frac{\left(\hat{\xi}_y^{(Y)}\right)^s \hat{\gamma}_s^{(Y)}}{\Gamma\left(\hat{\xi}_y^{(Y)}, \hat{\gamma}^{(Y)}\right)} \tag{6}$$

$$t = E\left(T \mid \hat{\xi}_t^{(T)}\right) = \sum_s s \frac{\left(\hat{\xi}_t^{(T)}\right)^s \hat{\gamma}_s^{(T)}}{\Gamma\left(\hat{\xi}_t^{(T)}, \hat{\gamma}^{(T)}\right)} \tag{7}$$

Calculating CML estimates of score parameters and finding the solutions to equations (5) - (7) are routine exercises for item analysis by Rasch models. DIGRAM uses iterative proportional fitting to calculate the CML estimates of score parameters and Newton-Raphson methods to solve equations (5)-(7).

3 Test equating by the Leunbach model

We refer to Holland and Dorans (2006) for an overview and a brief history of test equating. Test equating by the Leunbach model is an example of nonlinear True-Score equating.

Again, let X and Y be test scores of Test1 and Test2, respectively. Non-linear True-Score equating assumes that X and Y are two raw scores summarizing responses to sets of items from IRT models with a common latent variable θ.

In such models, true scores τ_X and τ_Y are the expected outcomes given θ,

$$\tau_X = \nu_X(\theta) = E(X|\theta) \text{ and } \tau_Y = \nu_Y(\theta) = E(Y|\theta) \tag{8}$$

The functions $\nu_X(\theta)$ and $\nu_Y(\theta)$ define the test characteristic curves of X and Y. They define a monotonic, but nonlinear symmetric relationship between the true scores given by

$$\tau_Y = \nu_Y\left(\nu_X^{-1}(\tau_X)\right) \text{ and } \tau_X = \nu_X\left(\nu_Y^{-1}(\tau_Y)\right) \tag{9}$$

Holland and Dorans (2006) suggest using the functions in (9) replacing true scores with observed scores

$$Y = \nu_Y\left(\nu_X^{-1}(X)\right) \text{ and } X = \nu_X\left(\nu_Y^{-1}(Y)\right) \tag{10}$$

The proposal is motivated by Hanson (1991) who showed that observed score equating by (10) is similar to true-score equating if the test characteristic curves were linear.

In Leunbach's model, test characteristic curves are defined by the expected outcomes of power series distributions. This connects test equating to IRT-base equating because raw scores from Rasch models and loglinear Rasch models with locally dependent items also have power series distributions.

The maximum likelihood estimates of the person parameters in the Leunbach model are equal to the person parameters where the expected value of the total score is the same as the observed score and therefore defined by $\nu_X^{-1}(x)$ and $\nu_Y^{-1}(y)$. We may therefor regard the observed score as

an unbiased ML estimate of the true score. For this reason, the proposal (10) of Holland and Dorans (2006) is justified even though the test characteristic curves are nonlinear.

The following pages show how to perform direct and indirect test equating in DIGRAM using data described below.

4 Data

The data for the example contains results for three sleep tests (Test1=ESS, Test2= MOS, and Test3=PSQI) from two datasets corresponding to two studies, TONiC (https://tonic.thewaltoncentre.nhs.uk/) and PROMIS (Pilkonis 2016) (http://www.healthmeasures.net/explore-measurement-systems/promis). ESS was common to both studies, MOS only available in TONiC, and PSQI only available in PROMIS. The following table describes the tests:

Test	Complete name	Number of items	Item (scale) range	Availability (sample size)
ESS (Sargento, Perea et al. 2015)	Epworth Sleepiness Scale	8	0-3 (0-24)	TONiC and PROMIS
MOS (Viala-Danten, Martin et al. 2008)	Medical Outcomes Study	6	0-4 (0-24)	TONiC (N=722)
PSQI (Buysse, Reynolds et al. 1989)	Pittsburgh Sleep Quality Index	12	0-3 (0-36)*	PROMIS (N=2974)

Table 1

*Only the categorical items of the PSQI were considered. The sum of the individual items instead of the existing algorithm was applied. The two items *How often have you taken medicine to help you sleep (prescribed or 'over the counter')? / Do you have a bed partner or roommate?)* were not considered in the summated score.

For DIGRAM to perform test equating, you have to create a DIGRAM project containing both data sets where PSQI is missing for the first 722 rows, and MOS is missing for the last 2974 rows. In the project that we have used to illustrate the analyses, the three tests are labelled A, B, and F.

In what follows, we first illustrate direct equating of ESS (A) and MOS (B). Then we show how to establish indirect equating of MOS (B) and PSQI (F) via ESS (A).

5 Direct test equating in DIGRAM

Use the **EQUATE** command to invoke test equating in DIGRAM and type the name of two or three variables to equate and a * if you want extended output where you can see what happens during the analysis in the following way:

**EQUATE A B**	invokes direct equating of A onto B and vice versa.
**EQUATE A B ***	invokes direct equating of A and B providing extensive and verbose output.

During initialization, DIGRAM reminds you that the two test scores do not have to be raw scores from a Rasch model.

```
  +---------------+
  |               |
  | Test equating |
  |               |
  +---------------+

Test scores:
    A - "ess"
    B - "mos"

Test scores are not subscales defined by a Rasch model

=========================================
```

ESS and MOS are available in the TONIC sample (N=722), but only complete cases (i.e., without missing values) are considered for the equating (671). The table on the next page shows that most of the observed counts are located in its 'diagonal'. Or alternatively, that the upper-right and lower-left triangles of this table are 'empty', which is a good sign for test equating to take place.

The two scores are positively correlated. Since the Leunbach model is nonlinear and since data is summarized in a table, DIGRAM measures the correlation by Goodman and Kruskal (1954), which is closely related to Kendall's Tau.

In this case, Gamma is equal to 0.293. The correlation cannot be described as impressive. However, before we dismiss the association as too weak to be caused by a common latent variable, we have to compare the observed correlation to the expected correlation under the Leunbach model. The result of this exercise will be shown later, after we have shown how to calculate the expected ESS-MOS counts under the model.

```
+------------------+
|                  |
| Observed counts  |
|                  |
+------------------+
```

A = "ess"
B = "mos"

	"mos"																						
"ess"	0	1	2	3	4	5	6	7	8	9	10	11	12	13	14	15	16	17	18	19	20	21	Total
0	5	6	4	4	2	2	4		1	3	1			1						1			34
1		3	6	3	2	2	3	2	2	1	1	2	2		1	2							32
2	1	1	3	5	5	2	6	1	5	1	7	2		4	3	1							47
3		1	6	3	4	4	3	5	4	5	1	4	4		3	1		2			1		51
4		3	6	5	4	9	7	3	4	5	2	1	5	1	2		1	2					60
5		2	2	3	2	4	4	3	3	2	1	2	2	1	4	2				1			38
6	3	3	2	3	3	4	4	5		7	5	3	1	1	1	3		1	1		1		51
7				1	1	7	7	2	3	5	3	4	7	4	5	1	1	1					52
8			1	2	2	1	6	3	2	3	4	3	3	2	2	2	2	2	1	1			42
9	1			1	2	4	6	5	2	6	1	1	3	2	1		3	1		2			41
10			1		1	1	1	3	5	4	2	2	2	3	1	2	1	1	1				31
11	1				1	1	2	1	3	2	3	2	4	3	1	1	2	1	2				30
12		1		1	3	2	2	1		1	1	4	6	4	2	2	1			1	1		33
13				1	3	1	1		2	3	1	3	1	1	3	3		1	2	2	1		29
14						3		1	1	1	1	2	1	1	1	5	1	1					19
15			1				1	2	1	1	3	2	3	4	1	1							20
16			2	1		3	1	2		3	1		1	2	1	1	2	1			1		22
17							1	1		1	1	1		1				1	1			1	9
18						1				1		2		2			2						8
19								2			1	1		1		1				1			7
20										1	1						1		1				4
21										1				1	1	1							4
22					1						1	2				1	1						6
23								1															1
Total	11	20	34	33	36	51	59	43	38	57	42	43	45	39	33	30	18	15	9	9	5	1	671

Table 2

The first step is to calculate the conditional maximum likelihood estimates of score parameters of ESS and MOS. The two tables below show the results.

```
671 persons included

Ranges:
          "ess":     0   23
          "mos":     0   21
Estimated score parameters:
```

A - "ess"	
0	1.00
1	1.73
2	3.85
3	5.56
4	8.43
5	6.78
6	10.72
7	12.78
8	11.87
9	12.73
10	10.32
11	10.81
12	12.32
13	11.27
14	7.47
15	7.94
16	8.75
17	3.50
18	3.00
19	2.48
20	1.44
21	1.37
22	2.06
23	0.31

B - "mos"	
0	1.00
1	4.80
2	14.07
3	20.06
4	30.65
5	56.55
6	82.07
7	74.40
8	75.62
9	128.70
10	106.47
11	119.77
12	132.01
13	121.96
14	106.92
15	99.07
16	57.48
17	49.63
18	29.99
19	29.36
20	15.95
21	3.18

Table 3

Next, DIGRAM uses the estimates of the score parameters of ESS and MOS to calculate estimates of the score parameters of T = ESS+MOS score. These estimates can be seen below.

T - "ess" + "mos"	
0	1.00
1	6.53
2	26.23
3	68.48
4	154.71
5	312.39
6	571.54
7	933.64
8	1453.56
9	2151.81
10	2933.26
11	3879.80
12	5038.17
13	6339.21
14	7486.51
15	8858.02
16	10088.91
17	11187.43
18	12228.17
19	12817.88
20	13126.45
21	13412.19
22	13184.23
23	12520.07
24	11875.73
25	11048.93
26	9906.73
27	8826.51
28	7652.55
29	6337.71
30	5232.64
31	4275.28
32	3268.23
33	2512.49
34	1874.48
35	1386.90
36	958.73
37	634.85
38	393.09
39	253.20
40	148.67
41	96.47
42	46.51
43	11.58
44	1.00

Table 4

Next, DIGRAM uses the estimates of the score parameters to calculate the expected counts under the Leunbach model and to test whether the model fits the data.

Leunbach (1976) referred to the set of outcomes with a given total score as an “orbit”. The orbit defined by a total score equal to 5 consists of the following combinations of ESS and MOS: (0,5), (1,4), (2,3), (3,2), (4,1), and (5,0). To calculate the expected counts on an orbit, DIGRAM first uses Formula (4) to calculate the conditional orbit probabilities given the orbit score, and then calculates the expected orbit count by multiplying the orbit probability with the number of persons on the orbit.

The next pages show the orbit probabilities and the expected orbit counts. The information on orbit 5 has been highlighted in red to make it more readable. The orbit probability of (ESS =1, MOS=4) is equal to 17.0 %. Since the total number of persons on this orbit is equal to 18 it follows that the expected orbit count is equal to 3.1.

To give a first impression of the degree to which the Leunbach model fits the data, DIGRAM next calculates and presents a table with standardized residuals. Since there is only a handful of residual below -1.96 or above 1.96, the results are promising. Unless one has a very large data set, one cannot really trust this impression. The expected values in most cells are small for which reason residuals do not have approximate normal distributions.

DIGRAM provides a solution to this problem. On each orbit, DIGRAM defines significant areas in the following way.

Let a and b be two scores taken by a person, t=a+b, and $p_{a|t}$ = Prob(A=a, B=b|T=t). To assess whether there is a significant difference between the two scores DIGRAM calculates the cumulative probabilities $pLow_{a|t} = \sum_{i=0}^{a} p_{a|t}$ and $pHigh_{a|t} = \sum_{i=a}^{t} p_{a|t}$ and conclude that “A<B” if $pLow_{a|t} \leq 0.05$ and that “A>B” if $pHigh_{a|t} \leq 0.05$.

The number of persons with significant differences between the two scores and each of these score combination can be found and will be commented upon on the page after the table with standardized residuals. However, since orbit distributions are discrete it is more complicated than that. For that reason, DIGRAM calculates the level of significance for each orbit and compares the observed numbers of cases with significant differences between the two test scores to the expected number of cases under the Leunbach model.

```
+-------------------------------+
|                               |
| Estimated orbit distributions |
|                               |
+-------------------------------+
```

A = "ess"
B = "mos"

A \ B	0	1	2	3	4	5	6	7	8	9	10	11	12	13	14	15	16	17	18	19	20	21
0	100.0	73.5	53.6	29.3	19.8	**18.1**	14.4	8.0	5.2	6.0	3.6	3.1	2.6	1.9	1.4	1.1	0.6	0.4	0.2	0.2	0.1	0.0
1	26.5	31.7	35.6	22.5	**17.0**	17.1	15.2	8.9	6.1	7.6	4.8	4.1	3.6	2.8	2.1	1.7	0.9	0.7	0.4	0.4	0.2	0.0
2	14.7	27.0	35.0	**24.7**	20.7	23.3	21.8	13.3	9.9	12.8	8.1	7.3	6.8	5.3	4.1	3.4	1.8	1.5	0.9	0.8	0.5	0.1
3	8.1	17.2	**25.0**	19.5	18.2	21.6	21.2	14.1	10.8	14.2	9.3	8.9	8.3	6.7	5.3	4.5	2.5	2.1	1.2	1.2	0.7	0.1
4	5.5	**13.0**	20.8	18.1	17.8	22.2	23.6	16.2	12.7	17.1	12.0	11.4	11.0	9.2	7.4	6.5	3.7	3.1	1.9	2.0	1.1	0.2
5	**2.2**	5.7	10.2	9.4	9.7	13.1	14.3	10.0	8.1	11.7	8.2	8.1	8.0	6.8	5.7	5.1	2.9	2.6	1.6	1.7	1.0	0.2
6	1.9	5.5	10.4	10.0	11.2	15.6	17.5	12.6	10.8	15.6	11.3	11.5	11.6	10.2	8.7	7.9	4.7	4.2	2.7	2.8	1.7	0.4
7	1.4	4.2	8.4	8.7	10.1	14.3	16.5	12.7	10.9	16.3	12.2	12.5	13.2	11.9	10.2	9.6	5.9	5.3	3.5	3.8	2.3	0.5
8	0.8	2.6	5.7	6.1	7.2	10.6	13.0	10.0	8.9	13.7	10.3	11.1	11.9	10.8	9.6	9.4	5.7	5.3	3.6	3.9	2.5	0.6
9	0.6	2.1	4.6	5.1	6.2	9.6	11.8	9.4	8.6	13.4	10.6	11.6	12.5	11.8	10.9	10.6	6.6	6.4	4.3	4.9	3.2	0.8
10	0.4	1.3	2.9	3.3	4.2	6.6	8.4	6.9	6.4	10.4	8.4	9.2	10.3	10.1	9.3	9.3	6.0	5.8	4.0	4.8	3.1	0.8
11	0.3	1.0	2.4	2.9	3.7	6.1	7.9	6.6	6.4	10.6	8.6	9.8	11.4	11.1	10.5	10.8	7.0	7.0	5.1	6.1	4.0	1.1
12	0.2	0.9	2.3	2.8	3.7	6.2	8.3	7.1	7.1	11.8	9.9	11.8	13.7	13.6	13.3	13.8	9.3	9.6	7.1	8.5	6.0	1.6
13	0.2	0.7	1.8	2.2	3.1	5.2	7.2	6.4	6.4	11.0	9.6	11.4	13.5	13.9	13.6	14.6	10.2	10.7	7.9	10.1	7.2	1.9
14	0.1	0.4	1.0	1.3	1.9	3.3	4.7	4.1	4.3	7.7	6.7	8.1	10.0	10.3	10.4	11.7	8.2	8.7	6.9	8.7	6.4	1.7
15	0.1	0.4	1.0	1.3	1.9	3.4	4.9	4.5	4.8	8.6	7.6	9.6	11.9	12.6	13.4	15.0	10.7	12.1	9.5	12.4	9.1	2.6
16	0.1	0.4	1.0	1.4	2.0	3.7	5.4	5.2	5.6	10.2	9.4	11.9	15.1	16.8	17.9	20.3	15.4	17.3	14.0	18.5	14.6	4.4
17	0.0	0.1	0.4	0.5	0.8	1.5	2.3	2.2	2.4	4.5	4.2	5.5	7.3	8.2	8.7	10.6	8.0	9.3	7.6	10.7	8.8	2.8
18	0.0	0.1	0.3	0.4	0.7	1.4	2.1	2.0	2.3	4.4	4.2	5.7	7.6	8.6	9.8	11.8	9.2	10.7	9.4	13.9	12.2	
19	0.0	0.1	0.3	0.4	0.6	1.2	1.8	1.9	2.1	4.2	4.2	5.7	7.7	9.3	10.6	13.1	10.3	12.9	11.7	18.6		
20	0.0	0.1	0.2	0.2	0.4	0.7	1.2	1.2	1.4	2.9	2.9	4.0	5.8	7.0	8.2	10.3	8.7	11.3	11.0			
21	0.0	0.0	0.2	0.2	0.4	0.8	1.3	1.3	1.6	3.4	3.4	5.0	7.2	8.9	10.6	14.2	12.4	17.3				
22	0.0	0.1	0.2	0.4	0.6	1.3	2.2	2.4	3.0	6.2	6.7	9.8	14.5	18.1	23.0	32.2	30.2					
23	0.0	0.0	0.0	0.1	0.1	0.2	0.4	0.4	0.6	1.2	1.3	2.0	3.0	4.0	5.3	7.9						

Table 5

```
+------------------+
|                  |
| Expected counts  |
|                  |
+------------------+
```

A = "ess"
B = "mos"

A \ B	0	1	2	3	4	5	6	7	8	9	10	11	12	13	14	15	16	17	18	19	20	21	TOTAL
0	5.0	4.4	4.3	3.2	1.8	**3.3**	3.6	1.5	1.1	1.4	1.0	0.6	0.9	0.6	0.3	0.4	0.2	0.1	0.1	0.1	0.0	0.0	34.0
1	1.6	2.5	3.9	2.0	**3.1**	4.3	2.9	2.0	1.5	2.1	1.0	1.5	1.1	0.6	0.7	0.6	0.3	0.2	0.1	0.1	0.1	0.0	32.0
2	1.2	3.0	3.2	4.5	5.2	4.4	4.8	3.2	2.8	2.7	2.9	2.2	1.4	1.8	1.4	1.0	0.5	0.4	0.2	0.2	0.1	0.0	47.0
3	0.9	1.6	**4.5**	4.9	3.5	4.8	5.1	3.9	2.3	5.1	2.8	1.9	2.7	2.4	1.5	1.4	0.7	0.4	0.3	0.2	0.2	0.0	51.0
4	0.5	**2.3**	5.2	3.4	3.9	5.3	6.6	3.4	4.6	5.1	2.5	3.8	3.9	2.7	2.2	2.0	0.7	0.9	0.3	0.5	0.2	0.1	60.0
5	**0.4**	1.4	1.9	2.1	2.3	3.7	3.0	3.6	2.4	2.4	2.7	2.8	2.3	2.0	1.7	0.9	0.8	0.4	0.4	0.4	0.2	0.0	38.0
6	0.5	1.0	2.3	2.4	3.1	3.3	6.3	3.8	2.3	5.1	4.0	3.3	3.5	3.1	1.6	2.2	0.8	1.0	0.6	0.6	0.3	0.1	51.0
7	0.3	0.9	2.0	2.4	2.1	5.2	5.0	2.7	3.6	5.7	3.5	3.8	3.9	2.1	2.9	1.6	1.4	1.1	0.7	0.6	0.3	0.1	52.0
8	0.2	0.6	1.6	1.3	2.6	3.2	2.7	3.3	3.1	4.0	3.1	3.3	2.1	3.0	1.6	2.3	1.2	1.1	0.6	0.6	0.4	0.1	42.0
9	0.1	0.6	1.0	1.8	1.8	2.0	3.9	3.3	2.5	4.0	3.2	2.1	3.5	2.0	2.6	2.2	1.4	1.0	0.6	0.7	0.4	0.1	41.0
10	0.1	0.3	1.0	1.0	0.9	2.2	2.9	2.0	1.9	3.1	1.5	2.6	1.8	2.4	2.0	1.9	1.0	0.9	0.6	0.7	0.3	0.1	31.0
11	0.1	0.4	0.7	0.6	1.2	2.1	2.3	2.0	1.9	1.9	2.4	1.7	2.7	2.3	2.2	1.7	1.1	1.1	0.7	0.5	0.3	0.1	30.0
12	0.1	0.3	0.5	0.9	1.3	1.8	2.5	2.1	1.3	3.3	1.7	2.8	2.9	2.9	2.1	2.1	1.4	1.4	0.6	0.6	0.4	0.1	33.0
13	0.1	0.2	0.6	0.8	0.9	1.6	2.2	1.1	1.8	1.9	2.3	2.4	2.8	2.2	2.0	2.2	1.4	1.0	0.6	0.7	0.3	0.1	29.0
14	0.0	0.1	0.4	0.4	0.6	1.0	0.8	1.2	0.7	1.8	1.4	1.7	1.6	1.5	1.6	1.6	0.7	0.6	0.5	0.3	0.3	0.0	19.0
15	0.0	0.1	0.3	0.4	0.6	0.6	1.4	0.8	1.2	1.8	1.6	1.5	1.8	1.9	1.9	1.4	0.7	0.8	0.4	0.6	0.2	0.1	20.0
16	0.0	0.1	0.3	0.4	0.4	1.0	0.9	1.2	1.2	2.1	1.5	1.8	2.3	2.4	1.6	1.4	1.1	0.7	0.7	0.4	0.4	0.0	22.0
17	0.0	0.0	0.1	0.1	0.2	0.3	0.6	0.5	0.5	0.7	0.6	0.8	1.0	0.7	0.6	0.7	0.3	0.5	0.2	0.3	0.1	0.1	9.0
18	0.0	0.0	0.1	0.1	0.1	0.3	0.4	0.4	0.4	0.7	0.6	0.8	0.7	0.6	0.7	0.5	0.5	0.2	0.3	0.1	0.5		8.0
19	0.0	0.0	0.1	0.1	0.1	0.2	0.4	0.3	0.3	0.6	0.6	0.5	0.5	0.6	0.4	0.7	0.2	0.4	0.1	0.7			7.0
20	0.0	0.0	0.0	0.1	0.1	0.2	0.2	0.2	0.2	0.4	0.3	0.3	0.4	0.3	0.4	0.2	0.3	0.1	0.4				4.0
21	0.0	0.0	0.0	0.0	0.1	0.1	0.2	0.2	0.2	0.3	0.2	0.4	0.3	0.4	0.2	0.4	0.1	0.7					4.0
22	0.0	0.0	0.1	0.1	0.1	0.2	0.3	0.3	0.3	0.4	0.5	0.4	0.7	0.4	0.7	0.3	1.2						6.0
23	0.0	0.0	0.0	0.0	0.0	0.0	0.1	0.0	0.0	0.1	0.1	0.1	0.1	0.1	0.1	0.3							1.0
TOT	11.0	20.0	34.0	33.0	36.0	51.0	59.0	43.0	38.0	57.0	42.0	43.0	45.0	39.0	33.0	30.0	18.0	15.0	9.0	9.0	5.0	1.0	671.0

40 iterative steps. Marginal Correction = 0.00008

Table 6

```
+------------------------+
|                        |
| Standardized residuals |
|                        |
+------------------------+

A = "ess"
B = "mos"
```

A \ B	0	1	2	3	4	5	6	7	8	9	10	11	12	13	14	15	16	17	18	19	20	21
0		0.8	-0.1	0.4	0.2	**-0.7**	0.2	-1.2	-0.1	1.3	-0.0	-0.8	-1.0	0.6	-0.5	-0.6	-0.4	-0.4	-0.3	3.6	-0.1	-0.1
1	-1.3	0.3	1.1	0.7	**-0.6**	-1.1	0.1	0.0	0.4	-0.8	0.0	0.4	0.9	-0.8	0.4	1.8	-0.5	-0.5	-0.3	-0.3	-0.2	-0.1
2	-0.2	-1.1	-0.1	**0.3**	-0.1	-1.2	0.6	-1.2	1.3	-1.0	2.4	-0.1	-1.2	1.7	1.3	0.0	-0.7	-0.7	-0.4	-0.5	-0.3	-0.2
3	-0.9	-0.4	**0.7**	-0.9	0.3	-0.3	-0.9	0.5	1.1	-0.0	-1.1	1.6	0.8	-1.5	1.2	-0.3	-0.9	2.6	-0.6	-0.5	2.0	-0.2
4	-0.7	**0.4**	0.4	0.8	0.0	1.6	0.2	-0.2	-0.3	-0.1	-0.3	-1.4	0.6	-1.0	-0.1	-1.4	0.4	1.2	-0.6	-0.7	-0.5	-0.2
5	**-0.6**	0.5	0.0	0.7	-0.2	0.2	0.6	-0.3	0.4	-0.3	-1.0	-0.5	-0.2	-0.7	1.8	1.1	-0.9	-0.7	-0.6	1.1	-0.5	-0.2
6	3.7	1.9	-0.2	0.4	-0.1	0.4	-0.9	0.6	-1.5	0.8	0.5	-0.2	-1.3	-1.2	-0.5	0.5	-0.9	-0.0	0.6	-0.8	1.4	-0.2
7	-0.5	-1.0	-1.4	-0.9	-0.8	0.8	0.9	-0.4	-0.3	-0.3	-0.3	0.1	1.5	1.3	1.3	-0.5	-0.3	-0.1	-0.9	-0.8	-0.6	-0.3
8	-0.4	-0.8	-0.5	0.6	-0.4	-1.2	2.0	-0.2	-0.6	-0.5	0.5	-0.2	0.6	-0.6	0.3	-0.2	0.7	0.8	0.6	0.5	-0.6	-0.3
9	2.3	-0.8	-1.0	-0.6	0.1	1.4	1.1	0.9	-0.3	1.0	-1.2	-0.8	-0.3	-0.0	-1.0	-1.5	1.4	-0.0	-0.8	1.5	-0.7	-0.3
10	-0.3	-0.5	-0.0	-1.0	0.1	-0.8	-1.1	0.7	2.2	0.5	0.4	-0.4	0.2	0.4	-0.7	0.0	0.0	0.1	0.5	-0.8	-0.5	-0.2
11	3.9	-0.6	-0.8	-0.8	-0.2	-0.8	-0.2	-0.7	0.8	0.1	0.4	0.3	0.8	0.4	-0.8	-0.6	0.9	-0.1	1.5	-0.7	-0.5	-0.3
12	-0.3	1.4	-0.7	0.1	1.5	0.1	-0.3	-0.8	-1.1	-1.3	-0.5	0.7	1.8	0.7	-0.1	-0.1	-0.3	-1.2	-0.8	0.5	0.9	-0.2
13	-0.2	-0.4	-0.8	0.2	2.2	-0.5	-0.8	-1.1	0.2	0.8	-0.9	0.4	-1.1	-0.8	0.7	0.5	-1.2	0.0	1.9	1.5	1.3	-0.3
14	-0.1	-0.4	-0.6	-0.6	-0.7	2.0	-0.9	-0.1	0.3	-0.6	-0.3	0.2	-0.5	-0.4	-0.5	2.6	0.3	0.5	-0.7	-0.6	-0.6	-0.2
15	-0.2	-0.4	1.3	-0.6	-0.8	-0.8	-0.3	1.4	-0.1	-0.6	1.1	0.4	0.9	1.5	-0.6	-0.3	-0.9	-0.9	-0.6	-0.8	-0.4	-0.3
16	-0.2	-0.3	3.1	0.9	-0.6	1.9	0.1	0.7	-1.1	0.6	-0.4	-1.3	-0.8	-0.2	-0.5	-0.4	0.9	0.4	-0.8	-0.6	0.9	-0.2
17	-0.1	-0.2	-0.3	-0.3	-0.5	-0.5	0.6	0.8	-0.7	0.3	0.5	0.2	-1.0	0.3	-0.8	-0.9	-0.6	0.8	2.2	-0.6	-0.3	2.6
18	-0.1	-0.2	-0.2	-0.4	-0.3	1.2	-0.7	-0.7	-0.6	0.4	-0.8	1.4	-0.8	1.8	-0.8	-0.7	2.3	-0.5	-0.5	-0.4	-0.7	
19	-0.1	-0.1	-0.3	-0.3	-0.4	-0.5	-0.6	3.1	-0.6	-0.8	0.5	0.7	-0.7	0.4	-0.7	0.4	-0.5	-0.6	-0.3	0.3		
20	-0.0	-0.1	-0.2	-0.2	-0.3	-0.4	-0.4	-0.4	-0.5	0.9	1.4	-0.5	-0.6	-0.5	-0.6	-0.5	1.5	-0.3	0.8			
21	-0.1	-0.1	-0.2	-0.2	-0.3	-0.4	-0.4	-0.4	-0.5	1.3	-0.5	-0.6	-0.5	0.8	1.7	0.9	-0.4	-0.8				
22	-0.1	-0.1	-0.2	-0.3	2.8	-0.4	-0.6	-0.6	-0.5	-0.7	0.8	2.6	-0.9	-0.6	-0.8	1.2	-0.2					
23	-0.0	-0.1	-0.1	-0.1	-0.1	-0.2	-0.2	4.8	-0.2	-0.3	-0.2	-0.3	-0.2	-0.3	-0.2	-0.6						

Table 7

```
+-----------------------------------------+
|                                         |
| Significant differences between A and B |
|                                         |
+-----------------------------------------+
```

A	B	n	p(A<B)	p(A>B)
0	10	1	0.0363	
0	13	1	0.0192	
0	19	1	0.0023	
1	14	1	0.0321	
1	15	2	0.0227	
2	15	1	0.0475	
3	17	2	0.0349	
3	20	1	0.0081	
5	19	1	0.0296	
6	0	3		0.0188
6	20	1	0.0194	
9	0	1		0.0059
10	2	1		0.0416
11	0	1		0.0028
12	1	1		0.0111
13	3	1		0.0375
15	2	1		0.0140
16	2	2		0.0117
16	3	1		0.0189
17	6	1		0.0472
17	21	1	0.0283	
18	5	1		0.0243
19	7	2		0.0455
22	4	1		0.0070
23	7	1		0.0045

```
31(4.6%) persons with significant differences between measurements

Confidence interval: [3.0,6.2]  Chi**2 =  0.2  df = 1   p =  0.6515
```

Table 8

DIGRAM finds 31 persons with test scores that are significantly different. Since this is comfortably within the confidence region defined by the Leunbach model, we find no reason to reject the hypothesis that ESS and MOS measure the same construct.

In addition to the person level assessments of differences in test scores, DIGRAM also calculates a likelihood ratio test comparing the observed and expected counts and a test comparing the observed and expected correlation between ESS and MOS.
The results are as follows:

```
LR =     382.39   DF = 426    p  =   0.936

Expected Gamma =   0.293  s.e. =  0.0266
Observed Gamma =   0.294    p  =  0.5117 (one-sided p.value)
```

The p-values are based on the asymptotic distributions of the test statistics. Because the ESS-MOS is a large sparse table, it is too optimistic to believe that these values can be trusted. For this reason, DIGRAM always suggests that you estimate the true p-values by parametric bootstrapping. You need to indicate the number of bootstrap samples in the following dialog box (1000 for this example):

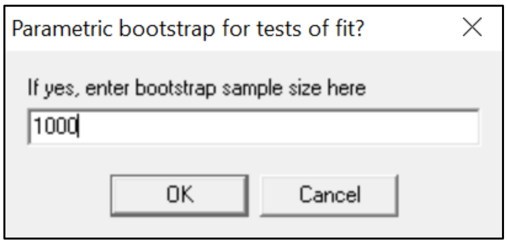

In this case the conclusion is the same. There is no evidence against the hypothesis that ESS and MOS measure the same construct.

```
  +---------------------------------------------------------+
  |                                                         |
  | Assessment of significance by parametric bootstrapping |
  |                                                         |
  +---------------------------------------------------------+

1000 bootstrap samples

Significance of the likelihood ratio test:  p = 0.588
Significance of gamma coefficient:          p = 0.602
```

Apart from the tests-of-fit, DIGRAM will only show you a little bit of the results on the previous pages unless you have asked for extended output. The following pages are more interesting since they show how to equate ESS scores to MOS and vice versa.

The table below shows how DIGRAM equates ESS scores onto MOS. The procedure is a three-step procedure:

The first step calculates the ML estimate of the person parameter given the ESS score. The estimate of a person parameter with ESS = 6 is equal to -0.2234093.

The second step calculates the expected MOS score for persons with person parameter equal to -0.2234093. In this case, the expected MOS score is 7.6.

Finally, the expected MOS score is rounded to give an equated MOS score as an integer score.

```
+----------------------------------+
|                                  |
| Equating of A-"ess" onto B-"mos" |
|                                  |
+----------------------------------+

 0       - inf        B =   0.0 ->    0
 1   -1.1983160  ->   B =   1.7 ->    2
 2   -0.7691466  ->   B =   3.0 ->    3
 3   -0.5471746  ->   B =   4.2 ->    4
 4   -0.4048681  ->   B =   5.4 ->    5
 5   -0.3026750  ->   B =   6.5 ->    7
 6   -0.2234093  ->   B =   7.6 ->    8
 7   -0.1582785  ->   B =   8.5 ->    9
 8   -0.1022868  ->   B =   9.4 ->    9
 9   -0.0523540  ->   B =  10.2 ->   10
10   -0.0064339  ->   B =  11.0 ->   11
11    0.0369408  ->   B =  11.7 ->   12
12    0.0789183  ->   B =  12.4 ->   12
13    0.1204938  ->   B =  13.0 ->   13
14    0.1626391  ->   B =  13.6 ->   14
15    0.2064325  ->   B =  14.2 ->   14
16    0.2532352  ->   B =  14.9 ->   15
17    0.3049920  ->   B =  15.5 ->   15
18    0.3648494  ->   B =  16.1 ->   16
19    0.4386756  ->   B =  16.8 ->   17
20    0.5397419  ->   B =  17.6 ->   18
21    0.7095011  ->   B =  18.5 ->   18
22    1.2098551  ->   B =  19.7 ->   20
23      + inf         B =  21.0 ->   21
```

Table 9

Equating of MOS onto ESS proceeds in exactly the same way. A MOS score equal to 8 gives an estimate of a person parameter equal to -0.1924633, an expected ESS score equal to 6.5 and an equated integer score equal to 6.

```
+----------------------------------+
|                                  |
| Equating of B-"mos" onto A-"ess" |
|                                  |
+----------------------------------+

 0       - inf    ->   A =   0.0 ->    0
 1   -1.6547931   ->   A =   0.5 ->    1
 2   -1.0527916   ->   A =   1.3 ->    1
 3   -0.7598416   ->   A =   2.0 ->    2
 4   -0.5773010   ->   A =   2.8 ->    3
 5   -0.4475849   ->   A =   3.7 ->    4
 6   -0.3472688   ->   A =   4.5 ->    5
 7   -0.2644907   ->   A =   5.5 ->    5
 8   -0.1924633   ->   A =   6.5 ->    6
 9   -0.1269384   ->   A =   7.5 ->    8
10   -0.0650038   ->   A =   8.7 ->    9
11   -0.0044409   ->   A =  10.0 ->   10
12    0.0566651   ->   A =  11.5 ->   11
13    0.1202192   ->   A =  13.0 ->   13
14    0.1884241   ->   A =  14.6 ->   15
15    0.2642457   ->   A =  16.2 ->   16
16    0.3523363   ->   A =  17.8 ->   18
17    0.4612698   ->   A =  19.3 ->   19
18    0.6102173   ->   A =  20.5 ->   20
19    0.8555799   ->   A =  21.5 ->   21
20    1.4437253   ->   A =  22.2 ->   22
21       + inf    ->   A =  23.0 ->   23
```

Table 10

Since percentile equating is a well-known and often-used method, extended output from DIGRAM also include this, so that percentile and Leunbach's equating can be compared.

The percentiles of ESS and MOS in the study population are shown below.

```
+-------------+
|             |
| Percentiles |
|             |
+-------------+
```

score	A	B
0	5.1	1.6
1	9.8	4.6
2	16.8	9.7
3	24.4	14.6
4	33.4	20.0
5	39.0	27.6
6	46.6	36.4
7	54.4	42.8
8	60.7	48.4
9	66.8	56.9
10	71.4	63.2
11	75.9	69.6
12	80.8	76.3
13	85.1	82.1
14	87.9	87.0
15	90.9	91.5
16	94.2	94.2
17	95.5	96.4
18	96.7	97.8
19	97.8	99.1
20	98.4	99.9
21	99.0	100.0
22	99.9	
23	100.0	

Table 11

Percentile equating searches for values of with percentiles as close to each other as possible and interpolates when percentiles disagree to some extent.

An A-score of 6 has a percentile value equal to 46.6 %. From this, it follows that ESS= 6 should be equated to a MOS score between 7 and 8 where percentiles are 42.8 % and 48.4%. Linear interpolation suggests that the equated MOS score should be 7.68, which rounds to an equated integer score equal to 8. Exactly the same as for equating by the Leunbach model.

```
+--------------------------------------------+
|                                            |
| Percentile equating of A-"ess" onto B-"mos" |
|                                            |
+--------------------------------------------+
```

A Score	A perc	equated interpolated	B score	B perc
0	5.1	1.09	1	4.6
1	9.8	2.03	2	9.7
2	16.8	3.42	3	14.6
3	24.4	4.59	5	27.6
4	33.4	5.66	6	36.4
5	39.0	6.42	6	36.4
6	46.6	7.68	8	48.4
7	54.4	8.70	9	56.9
8	60.7	9.60	10	63.2
9	66.8	10.56	11	69.6
10	71.4	11.27	11	69.6
11	75.9	11.93	12	76.3
12	80.8	12.77	13	82.1
13	85.1	13.61	14	87.0
14	87.9	14.20	14	87.0
15	90.9	14.87	15	91.5
16	94.2	16.00	16	94.2
17	95.5	16.60	17	96.4
18	96.7	17.22	17	96.4
19	97.8	18.00	18	97.8
20	98.4	18.44	18	97.8
21	99.0	18.89	19	99.1
22	99.9	20.00	20	99.9
23	100.0	21.00	21	100.0

Table 12

In percentile equating of MOS onto ESS, MOS=8 to ESS= 6. Again exactly the same as equating by the Leunbach model.

```
+--------------------------------------------+
|                                            |
| Percentile equating of B-"mos" onto A-"ess" |
|                                            |
+--------------------------------------------+
```

B		equated A		
Score	perc	interpolated	score	perc
0	1.6	0.00	0	5.1
1	4.6	0.00	0	5.1
2	9.7	0.97	1	9.8
3	14.6	1.68	2	16.8
4	20.0	2.41	2	16.8
5	27.6	3.35	3	24.4
6	36.4	4.53	5	39.0
7	42.8	5.49	5	39.0
8	48.4	6.23	6	46.6
9	56.9	7.40	7	54.4
10	63.2	8.41	8	60.7
11	69.6	9.61	10	71.4
12	76.3	11.09	11	75.9
13	82.1	12.31	12	80.8
14	87.0	13.68	14	87.9
15	91.5	15.18	15	90.9
16	94.2	16.00	16	94.2
17	96.4	17.75	18	96.7
18	97.8	19.00	19	97.8
19	99.1	21.17	21	99.0
20	99.9	22.00	22	99.9
21	100.0	23.00	23	100.0

Table 13

Applications of ESS distinguish between ESS scores from 0-13 with few problems, 13-15 with moderate problems, and 16-23 with severe problems. The first table below shows how the ESS categories equate onto MOS for both percentile and Leunbach equating, followed by a table where the persons included in the study sample are distributed across ESS categories and equated MOS categories. We leave it to the reader to decide whether they think the association is adequate.

```
  +----------------------------------------------------+
  |                                                    |
  | Equated "mos" groups corresponding to "ess" groups |
  |                                                    |
  +----------------------------------------------------+

                Cumulated      Equated B categories
   A    values  frequences   Percentiles    Leunbach´s model
----------------------------------------------------------
   1    0 -  12    80.8        0 -  13         0 -  12
   2   13 -  15    90.9       14 -  15        13 -  14
   3   16 -  23   100.0       16 -  21        15 -  21

  +-----------------------------------+
  |                                   |
  | Equated categories - model based |
  |                                   |
  +-----------------------------------+

A = "ess"    B = "mos"    3 categories

          "mos"
"ess"     0-12  13-14  15-21  Total
-----------------------------------
   0-12    437     52     53    542
  13-15     40     11     17     68
  16-23     35      9     17     61
  Total    512     72     87    671
-----------------------------------

Cohen`s kappa =   0.150
```

Table 14

Test equating is a statistical estimation procedure with the same kind of random error as all other statistical estimates. To assess this error DIGRAM computes the Standard Error of Equating (SEE) via bootstrapping from data. The SEE corresponds to the standard deviation of equated scores over hypothetical replications of an equating procedure in samples from a population of test takers (Kolen and Brennan 2004). For a score x_i of test A, the SEE of the equated score on test B, $\widehat{eq}_B(x_i)$, can be computed using the following formula

$$se[\widehat{eq}_B(x_i)] = \sqrt{var[\widehat{eq}_B(x_i)]} = \sqrt{E\{\widehat{eq}_B(x_i) - E[\widehat{eq}_B(x_i)]\}^2}$$

We calculated the replications of the equating procedure in S=1000 bootstrap samples. The SEE formula using boostrap samples is as follows:

$$\widehat{se}_{boot}[\hat{e}_B(x_i)] = \sqrt{\frac{\sum_s \{\hat{e}_{B_s}(x_i) - \hat{e}_{B.}(x_i)\}^2}{S-1}},$$

where

$$\hat{e}_{B.}(x_i) = \frac{\sum_s \hat{e}_{B_s}(x_i)}{S}$$

The results are summarized in the table below.

For each value of ESS (A), the table includes information on

1) The estimate of the equated score on MOS (B)
2) The average equated score in the 1000 bootstrap samples
3) The standard deviation of the equated score (i.e., the SEE) in the 1000 bootstrap samples
4) The relative frequencies of bootstrap samples where the equated score is 2 points or more below the estimate (-2+), 1 point below (-1), equal to the estimate (0), 1 point above (1), and 2 or more points above (2+).

On the bottom-left part of the table, the weighted average SEE value over all equated pairs is shown (0.39).

```
+-----------------------------------------+
|                                         |
| Bootstrap results for equating of A -> B |
|                                         |
+-----------------------------------------+
```

				Frequency of bootstrap errors				
A	estimate	expected B	SEE	-2+	-1	0	1	2+
0	0	0.00	0.00			100.0		
1	2	1.78	0.41		21.6	78.4		
2	3	2.98	0.18		2.8	96.6	0.6	
3	4	4.08	0.28		0.1	91.4	8.5	
4	5	5.32	0.47			67.7	32.3	
5	7	6.56	0.50		43.8	56.2		
6	8	7.63	0.48		37.4	62.6		
7	9	8.55	0.50		44.6	55.4		
8	9	9.35	0.48			65.5	34.5	
9	10	10.12	0.33		0.2	87.4	12.4	
10	11	11.00	0.23		2.6	94.8	2.6	
11	12	11.77	0.43		23.8	75.8	0.4	
12	12	12.34	0.47			66.5	33.5	
13	13	13.01	0.30		3.9	91.2	4.9	
14	14	13.69	0.49		32.0	66.9	1.1	
15	14	14.26	0.47		1.3	71.8	26.8	0.1
16	15	14.90	0.47		16.7	77.2	6.0	0.1
17	15	15.52	0.55		0.8	48.1	49.1	2.0
18	16	16.15	0.54		7.8	70.4	21.2	0.6
19	17	16.83	0.57	0.4	25.1	66.1	8.3	0.1
20	18	17.60	0.61	1.5	42.1	51.2	5.2	
21	18	18.50	0.62		2.8	48.6	44.8	3.8
22	20	19.87	0.78	1.4	33.7	41.6	23.3	
23	21	20.61	0.49	0.2	38.2	61.6		

Average SEE = 0.39

Table 15

There are two ways to assess the standard error of equating.

The first way is to look at probabilities describing the risk that the bootstrapped results depart from the results of the analysis of the observed data, because the bootstrap results estimate what might happen if we collected new data.

Since the equated score is the rounded value of the expected score on the second test we cannot expect the new sample to give exactly the same result. In Table 9, we saw that the expected MOS score for a ESS score of 7 is equal to 8.5 which results in a equated integer MOS score equal to 9. A little bit of random error, means that the probability of an integer score of 8 must be close to the probability of an integer score of 9. It follows that one must expect that a new sample can be expected to provide equated scores that differ with one point from the equated scores estimated by the current sample.

For this reason, it makes sense to assess the degree of error by an estimate of the probability that the equated scores from the bootstrap samples differ with more than one point and to require that this probability is not too large. If we insist that this probability may not be larger than 10 %, for the precision of the equating to be adequate[1], it follows that we can conclude that equating of ESS onto MOS is adequate.

Another option is to look at the standard error of equating (SEE). To do this we have to look at configurations of bootstrap errors that we regard as acceptable and calculate the SEE for those configurations.

The situation with ESS = 7, where very little error would result in either a -1 or a 0 the SEE would be 0.50. It therefore follows that we, due the rounding of the expected scores on Test2, have to say that SEE = 0.50 is acceptable. Using this criterion, we conclude that equating of ESS scores above 16 is less than satisfactory.

If we on the other hand say that a distribution with Prob(error = -2+) = 0.025, Prob(error = -1) = 0.316, Prob(error = 0) = 0.316, Prob(error = 1) = 0.316, and Prob(error = 2+) = 0.025 is close to the worst case that we would regard as acceptable, it follows that a SEE values smaller than or equal to 0.91 are acceptable while SEE values larger than 0.91 are less than satisfactory.

Using this criterion, we again conclude that equating of ESS on MOS is adequate.

[1] The 10 % is an arbitrary choice. The user has to decide on his own, what the probability should be.

6 Indirect test equating in DIGRAM

We illustrate indirect equating with data from the TONIC and PROMIS studies.

The first example equates MOS scores (B) from TONIC onto PSQI scores (F) from PROMIS mediated by ESS scores (A) that are recorded by both studies.

Use the **EQUATE** command with references to three variables to invoke indirect test equating in DIGRAM.

EQUATE B A F invokes indirect equating of B onto F mediated by A.

Indirect equating is a three-step procedure.

Step 1: Direct equating of MOS (B) onto ESS (A)
Step 2: Direct equating of ESS (A) onto PSQI (F)
Step 3: Indirect equating of B onto F using the results of steps 1 and 2.

Notice that for B onto A the TONIC sample is used (hence, N=671 complete cases), and for A onto F the PROMIS sample is used (hence, N= 2229 complete cases).

Output from the first two steps are exactly as shown in the previous section except that extensive output is not available during indirect equating. Here we only show the final tables with the equated scores.

```
+-----------------------------------+
|                                   |
| Equating of B-"mos" onto A-"ess" |
|                                   |
+-----------------------------------+
```

0	->	A =	0.0	->	0
1	->	A =	0.5	->	1
2	->	A =	1.3	->	1
3	->	A =	2.0	->	2
4	->	A =	2.8	->	3
5	->	A =	3.7	->	4
6	->	A =	4.5	->	5
7	->	A =	5.5	->	5
8	->	A =	6.5	->	6
9	->	A =	7.5	->	8
10	->	A =	8.7	->	9
11	->	A =	10.0	->	10
12	->	A =	11.5	->	11
13	->	A =	13.0	->	13
14	->	A =	14.6	->	15
15	->	A =	16.2	->	16
16	->	A =	17.8	->	18
17	->	A =	19.3	->	19
18	->	A =	20.5	->	20
19	->	A =	21.5	->	21
20	->	A =	22.2	->	22
21	->	A =	23.0	->	23

Table 16

```
+-----------------------------------+
|                                   |
| Equating of A-"ess" onto F-"psq"  |
|                                   |
+-----------------------------------+
```

0	->	F	=	0.0	->	0
1	->	F	=	0.7	->	1
2	->	F	=	1.4	->	1
3	->	F	=	2.3	->	2
4	->	F	=	3.5	->	4
5	->	F	=	5.3	->	5
6	->	F	=	7.4	->	7
7	->	F	=	9.7	->	10
8	->	F	=	12.0	->	12
9	->	F	=	14.0	->	14
10	->	F	=	15.9	->	16
11	->	F	=	17.6	->	18
12	->	F	=	19.2	->	19
13	->	F	=	20.7	->	21
14	->	F	=	22.1	->	22
15	->	F	=	23.4	->	23
16	->	F	=	24.8	->	25
17	->	F	=	26.0	->	26
18	->	F	=	27.2	->	27
19	->	F	=	28.4	->	28
20	->	F	=	29.6	->	30
21	->	F	=	30.9	->	31
22	->	F	=	32.2	->	32
23	->	F	=	33.7	->	34
24	->	F	=	35.0	->	35

Table 17

To equate a MOS score onto PSQI, DIGRAM first finds the expected ESS score corresponding to the MOS score. In most cases, this is not an integer. For this reason, DIGRAM finds the expected PSQI scores corresponding to the integer scores below and above the expected ESS score, interpolates to find the “expected” PSQI score for the MOS score, and finally converts that number to an equated integer score.

Assume for instance that MOS = 6 corresponding to an expected ESS equal to 4.5 (Table 16).

The expected ESS scores are equal to 3.5 and 5.3 for respectively ESS = 4 and ESS = 5 (Table 17). Interpolating between these two numbers gives an expected PSQI = (3.5+5.3)/2=4.4 and therefore an integer equated score equal to 4 as shown in the table below.

```
+--------------------------------+
|                                |
| Indirect equating B -> A -> F |
|                                |
+--------------------------------+
```

B	expected F	F
0	0.0	0
1	0.4	0
2	0.9	1
3	1.4	1
4	2.1	2
5	3.1	3
**6**	**4.4**	**4**
7	6.4	6
8	8.6	9
9	10.9	11
10	13.4	13
11	15.9	16
12	18.4	18
13	20.7	21
14	22.9	23
15	25.0	25
16	27.0	27
17	28.8	29
18	30.3	30
19	31.6	32
20	32.5	32
21	33.7	34

Table 18

Finally, DIGRAM assess the error associated with indirect equating by bootstrapping from data in exactly the same way as for direct equating. The next table shows the results. Notice that bootstrapping may fail to provide equated scores. In this example it happened for B = 20 in 0.5 % of the bootstrapped samples and in 38.4 % of the samples for B = 21 because of the low frequency of persons with these scores in the data.

```
+---------------------------------------------------+
|                                                   |
| Bootstrap results for indirect equating of B -> F |
|                                                   |
+---------------------------------------------------+
```

				Frequency of bootstrap errors				
B	estimate	expected F	SEE	-2+	-1	0	1	2+
0	0	0.00	0.00			100.0		
1	0	0.05	0.22			95.0	5.0	
2	1	1.00	0.06		0.4	99.6		
3	1	1.27	0.45			72.6	27.4	
4	2	2.04	0.19			96.2	3.8	
5	3	3.05	0.28		1.4	91.8	6.8	
6	4	4.41	0.52		0.7	58.1	40.6	0.6
7	6	6.19	0.57		7.6	66.4	25.1	0.9
8	9	8.42	0.67	6.2	49.1	41.0	3.6	0.1
9	11	10.93	0.70	1.5	23.1	56.8	17.8	0.8
10	13	13.48	0.73	0.1	7.5	43.2	43.0	6.2
11	16	15.97	0.76	2.0	23.6	51.0	22.2	1.2
12	18	18.39	0.81	0.8	9.8	47.4	34.0	8.0
13	21	20.73	0.91	6.7	34.0	40.8	16.2	2.3
14	23	23.02	1.06	5.6	26.3	36.3	25.4	6.4
15	25	25.18	1.21	6.3	23.1	32.8	25.6	12.2
16	27	27.12	1.30	8.5	23.7	31.7	22.3	13.8
17	29	28.80	1.35	14.9	28.5	28.1	17.9	10.6
18	30	30.20	1.38	10.2	19.8	30.3	22.9	16.8
19	32	31.36	1.35	25.6	28.1	26.9	13.8	5.6
20	32	32.35	1.36	9.6	15.6	27.6	24.8	22.3
21	34	32.84	1.30	37.0	25.4	32.1	5.5	

Average SEE = 0.67

Table 19

We finally present a comparison of two ways of equating ESS onto MOS, (1) directly, and (2) indirectly mediated by the Neurological Sleep Index – Diurnal Sleepiness (NSD) scale. The latter is available in TONiC, consists of 16 items, and has a score range of 0-48.

The TONiC data includes 671 persons with ESS and MOS, 625 persons with ESS and NSD, and 632 persons with MOS and NSD. The table below shows the results. The few cases where direct and indirect equating disagrees have been highlighted.

	Equated MOS scores	
ESS	Direct	Indirect
0	0	0
1	2	2
2	3	3
**3**	**4**	**5**
**4**	**5**	**6**
5	7	7
6	8	8
7	9	9
8	9	9
9	10	10
10	11	11
11	12	12
12	12	12
13	13	13
13	14	14
15	14	14
16	15	15
**17**	**15**	**16**
**18**	**16**	**17**
19	17	17
20	18	18
**21**	**18**	**19**
22	20	20
23	21	21
Average SEE	0.39	0.47

Table 20

The comparison of the directly and indirectly MOS scores is encouraging. There are few and inconsequential differences between the scores. The average SEE of the indirectly equated scores are larger than for the directly equated, but that was to be expected because the indirect method combines two set of directly equated scores with errors, whereas the direct method only has one set.

6 References

Andersen, E. B. (1970). "Asymptotic Properties of Conditional Maximum-Likelihood Estimators." Journal of the Royal Statistical Society. Series B (Methodological) **32**(2): 283-301.

Buysse, D. J., C. F. Reynolds, 3rd, et al. (1989). "The Pittsburgh Sleep Quality Index: a new instrument for psychiatric practice and research." Psychiatry Res **28**(2): 193-213.

Goodman, L. A. and W. H. Kruskal (1954). "Measures of Association for Cross Classifications " J.Amer.Statist.Assoc. **49**: 732-764.

Hanson, B. A. (1991). "A note on Levine's formula for equating unequally reliable tests using data from the common item nonequivalent groups design." Journal of Educational Statistics **16**(2): 93-100.

Holland, P. W. and N. J. Dorans (2006). Linking and Equating. Educational Measurement. R. L. Brennan, Praeger Publishers.

Kolen, M. J. and R. L. Brennan (2004). Test Equating, Scaling, and Linking. Methods and practices (2nd edition). New York, Springer.

Kreiner, S. and M. Mesbah (2013). Rasch Models for Ordered Polytomous Items. Rasch Models in Health. K. B. Christensen, S. Kreiner and M. Mesbah, Wiley: 27-41.

Kreiner, S. and T. Nielsen (2013). Item analysis in DIGRAM 3.04. Part I: Guided tours. Research report 2013/06, University of Copenhagen, Department of Public Health.

Leunbach, G. (1976). A probabilistic measurement model for assessing whether two tests measure the same personal factor Copenhagen, Danish Institute for Educational Research.

Noack, A. (1950). "A Class of Random Variables with Discrete Distributions." The Annals of Mathematical Statistics **21**(1): 127-132.

Pilkonis, P. (2016). PROMIS 2 Sleep Wake, Harvard Dataverse.

Rasch, G. (1960). Probabilistic Models for Some Intelligence and Attainment Tests, Danmarks Paedagogiske Institut.

Sargento, P., V. Perea, et al. (2015). "The Epworth Sleepiness Scale in Portuguese adults: from classical measurement theory to Rasch model analysis." Sleep Breath **19**(2): 693-701.

Viala-Danten, M., S. Martin, et al. (2008). "Evaluation of the reliability and validity of the Medical Outcomes Study sleep scale in patients with painful diabetic peripheral neuropathy during an international clinical trial." Health Qual Life Outcomes **6**: 113.
